# Neuroligin-3 confines AMPA receptors into nanoclusters, thereby controlling synaptic strength at the calyx of Held synapses

**DOI:** 10.1126/sciadv.abo4173

**Published:** 2022-06-15

**Authors:** Ying Han, Ran Cao, Liming Qin, Lulu Y. Chen, Ai-Hui Tang, Thomas C. Südhof, Bo Zhang

**Affiliations:** 1 School of Chemical Biology and Biotechnology, Peking University Shenzhen Graduate School, Shenzhen 518055, China.; 2 Institute of Neurological and Psychiatric Disorders, Shenzhen Bay Laboratory, Shenzhen 518132, China.; 3 Institute of Artificial Intelligence, Hefei Comprehensive National Science Center, Hefei 230026, China.; 4 CAS Key Laboratory of Brain Function and Disease, Ministry of Education Key Laboratory for Membrane-less Organelles & Cellular Dynamics and Hefei National Laboratory for Physical Sciences at the Microscale, Division of Life Sciences and Medicine, University of Science and Technology of China, Hefei 230026, China.; 5 Department of Anatomy and Neurobiology, University of California, Irvine, Irvine, CA 92697, USA.; 6 Department of Molecular and Cellular Physiology, Howard Hughes Medical Institute, Stanford University School of Medicine, Stanford, CA 94043, USA.

## Abstract

The subsynaptic organization of postsynaptic neurotransmitter receptors into nanoclusters that are aligned with presynaptic release sites is essential for the high fidelity of synaptic transmission. However, the mechanisms controlling the nanoscale organization of neurotransmitter receptors in vivo remain incompletely understood. Here, we deconstructed the role of neuroligin-3 (Nlgn3), a postsynaptic adhesion molecule linked to autism, in organizing AMPA-type glutamate receptors in the calyx of Held synapse. Deletion of *Nlgn3* lowered the amplitude and slowed the kinetics of AMPA receptor–mediated synaptic responses. Super-resolution microscopy revealed that, unexpectedly, these impairments in synaptic transmission were associated with an increase in the size of postsynaptic PSD-95 and AMPA receptor nanoclusters but a decrease of the densities in these clusters. Modeling showed that a dilution of AMPA receptors into larger nanocluster volumes decreases synaptic strength. Nlgn3, likely by binding to presynaptic neurexins, thus is a key organizer of AMPA receptor nanoclusters that likely acts via PSD-95 adaptors to optimize the fidelity of synaptic transmission.

## Teaser

Nlgn3 regulates the distribution of AMPA receptors at the calyx of Held synapse.

## INTRODUCTION

AMPA-type glutamate receptors (AMPARs), composed of GluA1 to GluA4 proteins, generate fast synaptic currents at most excitatory synapses in the mammalian brain (*1*, *2*). Recent studies using super-resolution optical imaging and electron microscopy demonstrated that glutamate receptors are clustered in nanodomains within the postsynaptic density (PSD) (*3*–*8*). Computational models predict that both the amplitude and the reliability of synaptic responses are improved if AMPARs are organized into nanoclusters that are aligned with presynaptic glutamate release sites (*9*–*11*), and that synaptic responses become weaker and more variable if AMPAR clusters are disorganized (*9*, *12*). Native AMPARs consist of homo- and heteromeric combinations of four GluA1 to GluA4 subunits associated with diverse ancillary cofactors, generating a tremendous heterogeneity in gating kinetics in different brain regions. In particular, GluA1/2-containing AMPARs exhibit slower gating properties than GluA3/4-containing AMPARs (*13*). Genetic evidence demonstrates that deletion of various AMPARs reduces the miniature excitatory postsynaptic current (mEPSC) amplitude and alters the mEPSC kinetics in different brain regions, consistent with the differential expression of various GluA isoforms (*14*, *15*).

The maintenance of the content and distribution of postsynaptic AMPARs relative to presynaptic release sites is thought to determine the efficiency of excitatory synaptic transmission. The well-matched pre- and postsynaptic specializations of a synapse are likely mediated by trans-synaptic cell adhesion molecules, including neurexins and neuroligins (Nlgns) that bind to each other (*16*). Mutations of *Nlgn* genes have been repeatedly linked to autism spectrum disorder (*17*–*20*). Four *Nlgn* genes (*Nlgn1* to *Nlgn4*) are expressed differentially in different vertebrate cells (*16*, *21*–*26*). Extensive studies using genetic deletions of *Nlgns* in mice suggested that deletion of *Nlgns* weakens excitatory and inhibitory synaptic transmission in different neuronal circuits (*27*–*35*). Overexpressing *Nlgn1* in hippocampal cultured neurons increased the density of postsynaptic AMPARs and enhances AMPAR-mediated synaptic transmission (*36*). Moreover, deletion of *Nlgn1* decreased the amplitude of AMPAR-mediated synaptic responses in cultured neurons (*37*, *38*), although no such decrease was observed in hippocampal brain slices (*28*, *32*).

To explore the mechanisms that organize AMPARs at postsynaptic sites, we used region- and cell type–specific *Nlgn3* conditional knockout (cKO) mice, brain slice electrophysiology, super-resolution microscopy, and computational modeling. We found that Nlgn3 is critical for the organization of precise AMPAR nanoclusters, suggesting that the loss of the organizational function of Nlgn3 could account, at least in part, for the pathophysiological changes observed with *NLGN3* mutations in human patients.

## RESULTS

### Deletion of *Nlgn3* impairs excitatory synaptic transmission at the calyx of Held

Postsynaptic neurons in the medial nucleus of the trapezoid body (MNTB) that are the targets of the calyx of Held synapses largely lack dendrites, rendering them well suited for electrophysiological voltage-clamp recordings with a high signal-to-noise ratio (*39*). The quantal size of evoked EPSCs at calyx synapses is nearly identical to the amplitude of mEPSCs, with a narrow amplitude size distribution for both types of signals that shift to larger values during synapse maturation (*40*). Most synaptic currents at the calyx of Held synapse during a quantal event is mediated by AMPARs that are clustered into postsynaptic “hotspots” with a few hundred nanometers radius centered around the presynaptic vesicle release site (*41*). At the calyx synapse, the amplitude and kinetics of mEPSCs are fine-tuned by the glutamate concentration in the synaptic cleft (*39*) and the content and properties of postsynaptic receptors (*15*). Therefore, mEPSCs are a reliable indicator of the properties of postsynaptic AMPARs at the calyx synapse.

**Fig. 1.**
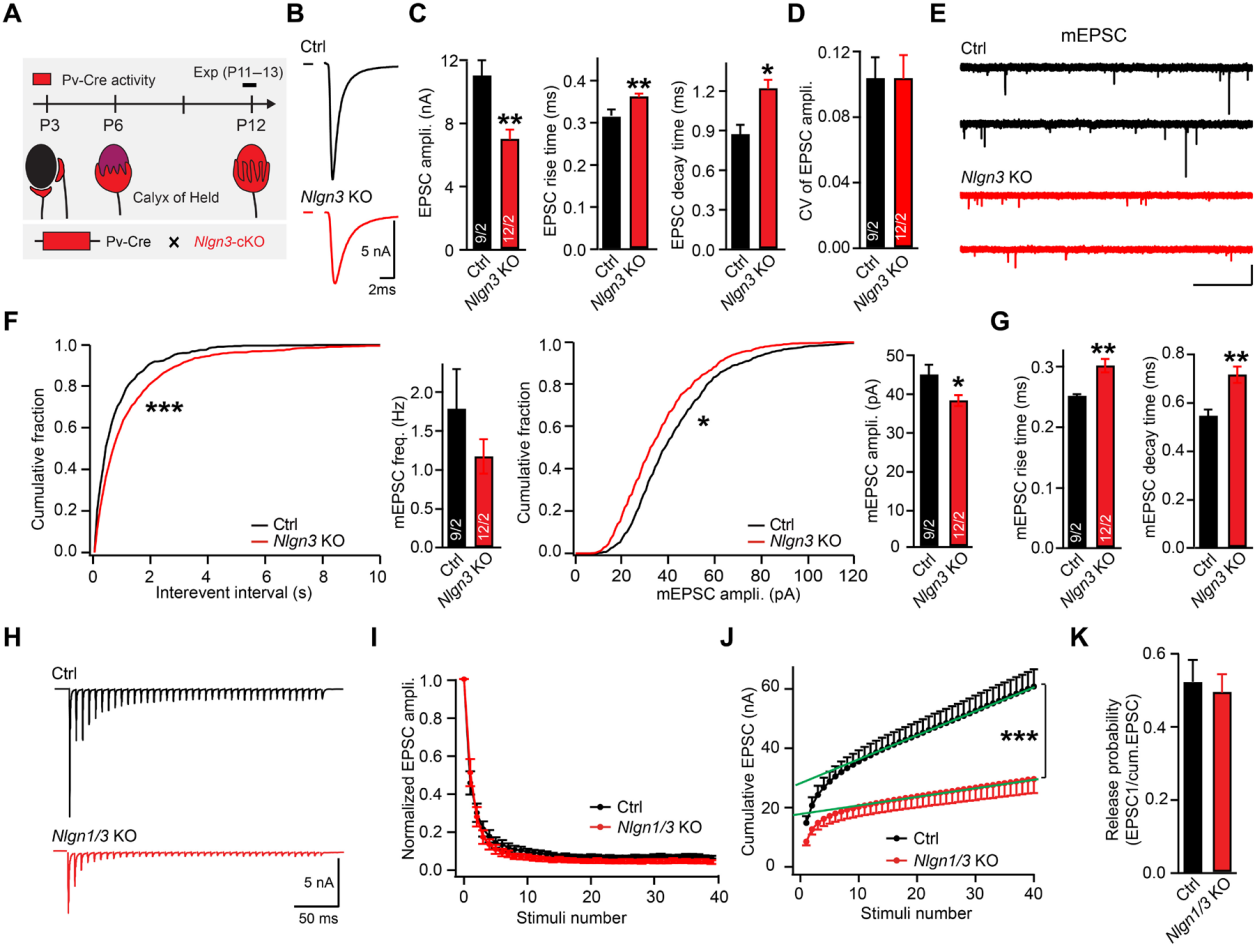
Deletion of *Nlgn3* reduces the strength and speed of excitatory synapses at the calyx of Held. Data were collected from patch-clamp recordings in acute slices from mice at P12/13. (**A**) Schematic of the strategy of the conditional *Nlgn3* deletion from the calyx of Held synapse. (**B**) Example traces of AMPAR-mediated EPSCs were recorded in response to afferent fiber stimulation from MNTB neurons (control, black; *Nlgn3* KO, red). (**C**) Analyses of the EPSC amplitude (left), rise (middle), and decay times (right). (**D**) Analyses of the coefficient of variation of EPSC in (B). (**E**) Example traces of AMPAR-mediated mEPSCs were recorded from MNTB neurons (control, black; *Nlgn3* KO, red). (**F** and **G**) Analyses of the mEPSC frequency and amplitudes (F) and of the rise and decay times (G). (**H**) Example traces of AMPAR-mediated EPSCs evoked by an action potential train (40 action potentials at 100 Hz; control, black; *Nlgn1/3* KO, red). (**I** and **J**) Analyses of the normalized (I) and cumulative EPSC amplitudes (J). (**K**) Analysis of the release probability as indirectly calculated by the ratio of the first EPSC to the cumulative EPSC amplitudes. Data are means ± SEM. Numbers in bars represent the numbers of cells/animals. Statistical significance was determined by a two-tailed Student’s *t* test (C, D, F, G, and K), by single-factor ANOVA (I and J), or by the Kolmogorov-Smirnov test (F), with *P < 0.05, **P < 0.01, and ***P < 0.001.

Phase-locked high-fidelity synaptic transmission at the calyx of Held is observed at postnatal day 8 (P8) to P9 (*42*) and becomes mature by P12 to P14 when functional hearing develops. We generated *Nlgn3* KO mice by crossing mice expressing Cre-recombinase under control of the parvalbumin (Pv) promoter (*33*) with mice carrying loxP-flanked *Nlgn3* alleles (Fig. 1A). Consistent with previous results (*33*), the *Nlgn3* deletion robustly decreased the amplitude of evoked EPSCs (~35%) at P12 to 13 calyx synapses and increased the EPSC rise (~15%) and decay times (~30%) (Fig. 1, A to C). Moreover, the *Nlgn3* deletion reduced the frequency (~30%) and amplitude of mEPSCs (~10%) and also prolonged their rise (~15%) and decay times (~20%; Fig. 1, E to G). Similar to our previous results from *Nlgn3* KO mice (*33*), we found that deletion of *Nlgn1* and *Nlgn3* caused a major decline in the cumulative EPSC amplitude during a high-frequency stimulus train (Fig. 1, H to J, and fig. S1C), suggesting a reduction in the size of the presynaptic readily releasable pool. However, we detected no change in short-term synaptic plasticity assessed as the ratio of the cumulative to the initial EPSC amplitude (Fig. 1, I to K) and the coefficient of variation of EPSCs (Fig. 1D), suggesting that the release probability was unaltered. Therefore, the reduction of synaptic transmission by the *Nlgn3* deletion likely arises from postsynaptic changes.

*Nlgn1* is also expressed at excitatory synapses (*43*), suggesting that *Nlgn1* might additionally regulate AMPAR-mediated release. To test this possibility, we injected lentiviruses coexpressing Cre-recombinase and green fluorescent protein (GFP) into the MNTB of *Nlgn1* cKO mice at P0 and analyzed these mice at P8 to P9 (fig. S1A). As a control, we injected littermates with lentiviruses expressing only GFP. The *Nlgn1* deletion reduced *N*-methyl-d-aspartate receptor (NMDAR)–mediated, but not AMPAR-mediated, EPSCs (fig. S1B), suggesting that *Nlgn1* does not perform an essential role on AMPAR-mediated EPSCs in the calyx synaptic transmission. However, the role of *Nlgn1* could be occluded by a more decisive function of *Nlgn3*, and the residual calyx synapse EPSC in *Nlgn3* KO mice could be maintained by *Nlgn1*. To assess this possibility, we generated *Nlgn1*/3 double cKO mice containing the Pv-Cre allele (fig. S1C). Unexpectedly, Cre-mediated deletion of both *Nlgn1* and *Nlgn3* in the MNTB produced the same extent of synaptic calyx phenotype as the *Nlgn3* deletion alone (fig. S1, D to I). Thus, Nlgn1 is dispensable for AMPAR-mediated transmission at the calyx of Held synapses, consistent with studies on hippocampal synapses at which Nlgn1 makes a major contribution to NMDAR-mediated (but not AMPAR-mediated) synaptic transmission (*28*, *32*). Together, these data indicate that *Nlgn3* plays an essential role in synaptic transmission at the calyx of Held such that its deletion decreases the postsynaptic glutamate receptor response without altering the presynaptic release probability.

### The *Nlgn3* deletion does not generally disrupt the architecture of calyx synapses

During synaptic transmission, postsynaptic responses to a given amount of released neurotransmitters depend on both the receptor numbers and the trans-synaptic release-receptor alignment (*8*, *44*). Specifically, while changes in receptor numbers alter mEPSCs and evoked EPSCs evenly, the intrasynaptic distribution of postsynaptic receptors relative to presynaptic release sites preferentially affects evoked EPSCs (*2*, *3*, *7*). The *Nlgn3* deletion resulted in a larger reduction in the amplitudes of evoked EPSCs than of mEPSCs (Fig. 1), suggesting a possible impairment in the nanoscale release-receptor alignment in the calyx synapse. To test this hypothesis, we used three-dimensional stochastic optical reconstruction microscopy (3D STORM) imaging (*8*, *45*) that allowed us to quantitatively analyze the nanoscale organization of key synaptic proteins.

**Fig. 2.**
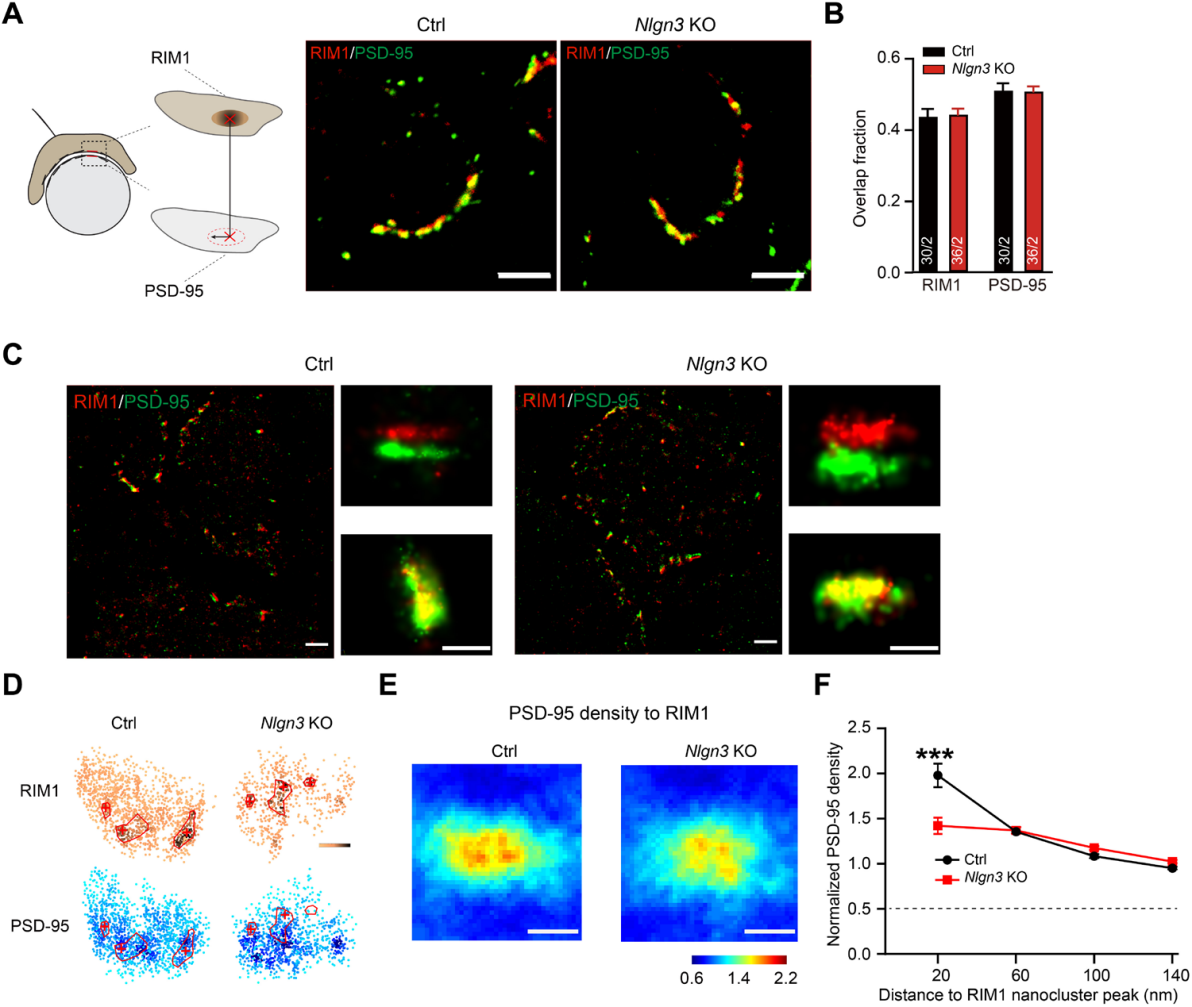
Decreased local enrichment of PSD-95 after deletion of *Nlgn3* at the calyx of Held. Data were collected from mice at P12. (**A**) Example confocal images of double staining of RIM1 (red) and PSD-95 (green) from P12 MNTB brain slices of control (left) and *Nlgn3* KO (right) mice. Scale bars, 5 μm. (**B**) Analysis of overlap fraction of RIM1 and PSD-95 with confocal images from P12 MNTB brain slices. (**C**) Representative images of dual-color 3D STORM from P12 MNTB brain slices and example individual synapses for the enlarged figures, side and en face views for the top and bottom panels, respectively. Scale bars, 2 μm for the broad field and 0.5 μm for the individual synapses. (**D**) Distributions of synaptic RIM1 and PSD-95 for control and *Nlgn3* KO mice. Red circles indicate nanoclusters, and red pluses denote the center of RIM1 clusters. (**E**) Representation of PSD-95 density across from RIM1 peak density averaged across synapses. Scale bars, 100 nm. (**F**) Quantification of normalized PSD-95 density as a function of the distance to the RIM1 nanocluster center (*n* > 170 clusters per three mice). Data are means ± SEM. Numbers in bars represent the number of synapses/animals. Statistical significance was determined by a two-tailed Student’s *t* test (B) or by the multiple unpaired *t* tests (F), with ***P < 0.001.

Because of the lack of efficient pan-AMPAR antibodies for STORM imaging, we assessed the trans-synaptic nanoalignment of release sites and receptors at the calyx synapse by imaging the presynaptic and postsynaptic scaffolding proteins Rab3-interacting molecule 1 (RIM1) and PSD-95. RIM1 organizes the nanoscale organization of the presynaptic active zone (*8*, *46*), while PSD-95 colocalizes with postsynaptic AMPAR nanoclusters (*4*, *6*). Confocal imaging uncovered no marked changes in RIM1 or PSD-95 staining and or in the degree of overlap between RIM1 and PSD-95 (Fig. 2, A and B). STORM imaging showed that both RIM1 and PSD-95 were assembled in disc-shaped clusters, which we refer to as synaptic clusters (Fig. 2, C and D). The distribution of RIM1 and PSD-95 within these clusters was not uniform but exhibited one to three notable high-density peaks, called subsynaptic nanoclusters, that we quantified using an algorithm based on local densities (*47*). To measure the trans-synaptic nanoalignment of RIM1 and PSD-95, we quantified the normalized relative local density of PSD-95 as a function of the distance from the peak of RIM1 nanoclusters in the en face plane (Fig. 2, D to F) (*7*, *8*, *47*). Whereas in control calyx synapses we detected a good alignment of presynaptic RIM1 nanoclusters with postsynaptic PSD-95 nanoclusters, we found that in *Nlgn3*-deficient synapses the normalized density of PSD-95 at regions opposite to the center (<60-nm radius) of RIM1 nanoclusters was significantly reduced (Fig. 2, D to F). Together, these results suggest that the deletion of *Nlgn3* decreases the recruitment of postsynaptic PSD-95 nanoclusters to presynaptic neurotransmitter release sites.

### The *Nlgn3* deletion increases the size of PSD-95 nanoclusters

**Fig. 3.**
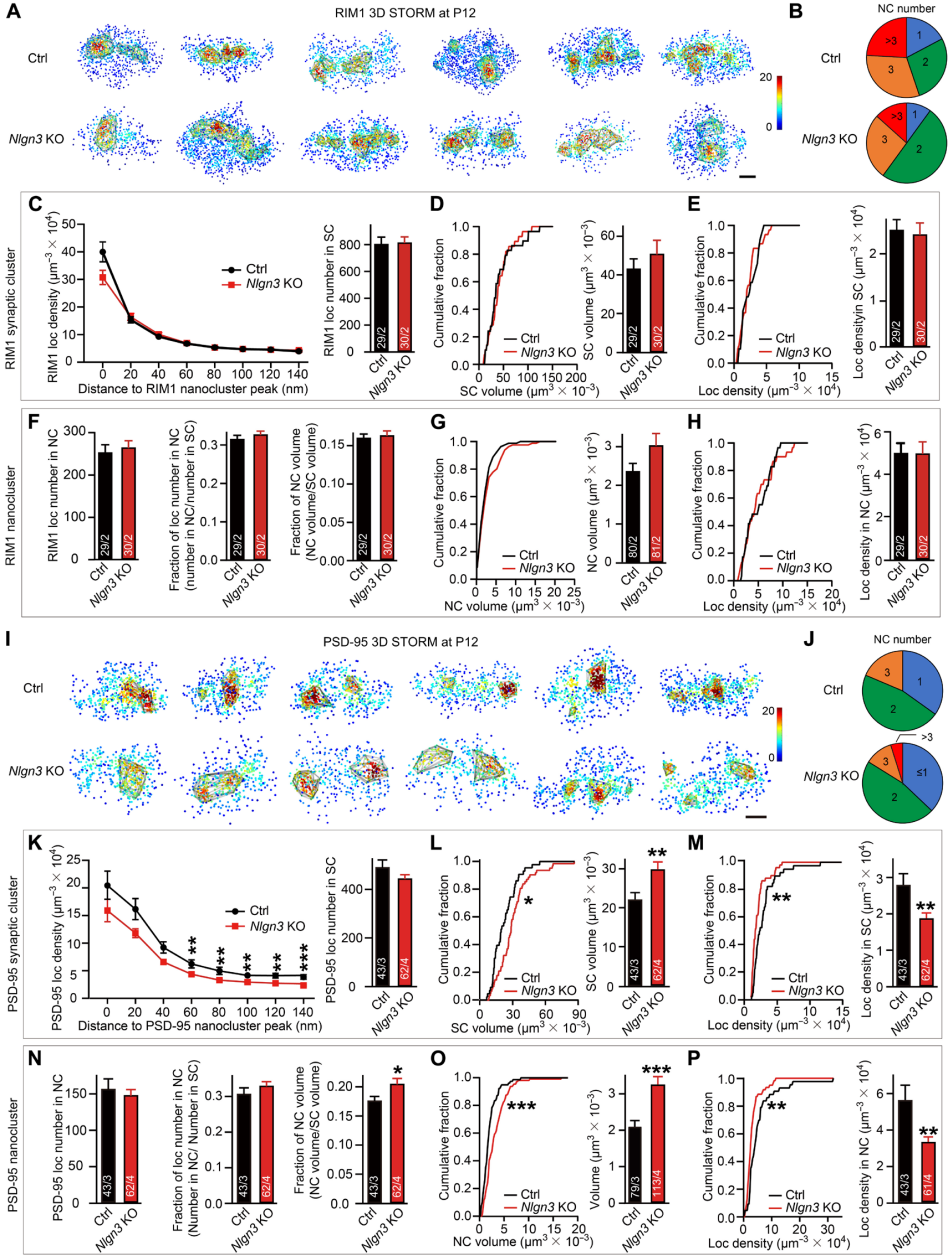
The *Nlgn3* deletion causes an increase in the size of PSD-95 nanoclusters and decreases the density of PSD-95 signal in these nanoclusters with normal RIM1 nanoclusters. Data were collected from mice at P12. (**A**) Example en face views of synaptic RIM1 local density maps in P12 MNTB neurons of control and *Nlgn3* KO mice. The gray lines depict the RIM1 nanocluster. Scale bar, 100 nm. (**B**) Pie charts show the roughly similar percentage of the RIM1 nanocluster number per synapse at P12 in both groups. (**C**) Analyses of RIM1 local density as a function of the distance to the RIM1 nanocluster peak. (**D** and **E**) Analyses of RIM1 synaptic cluster volume (D) and local density in the synaptic cluster (E). (**F**) Analysis of RIM1 nanocluster. RIM1 local number (left), the fraction of RIM1 local number (middle), and the fraction of RIM1 nanocluster volume (right). (**G** and **H**) Analyses of RIM1 nanocluster volume (G) and local density in nanocluster (H). (**I**) Example en face views of PSD-95 local density maps in P12 MNTB neurons of control and *Nlgn3* KO mice. The gray lines depict the PSD-95 nanocluster. Scale bar, 100 nm. (**J** to **P**) Similar to (B) to (H) but with PSD-95 clusters. Data are means ± SEM. Numbers in bars represent the number of synapses/animals. Statistical significance was determined by a two-tailed Student’s *t* test (C to H and K to P), by the Kolmogorov-Smirnov test (D, E, G, H, L, M, O, and P), by the multiple unpaired *t* tests (C and K), or by the Mann-Whitney *U* test (B and J), with *P < 0.05, **P < 0.01, and ***P < 0.001.

The decrease in the local enrichment of PSD-95 opposite to RIM1 nanoclusters could arise from a misalignment of pre- and postsynaptic nanoclusters and/or an alteration of the postsynaptic nanocluster organizations per se. To tease apart these possibilities, we examined the properties of synaptic clusters and subsynaptic nanoclusters of both proteins after the deletion of *Nlgn3*. We found that the *Nlgn3* deletion had no major effect on the properties of either RIM1 synaptic clusters or subsynaptic nanoclusters, including their localization numbers, volumes, and densities, nor did it change the number of RIM1 nanoclusters per synaptic cluster (Fig. 3, A to H). Thus, consistent with the normal release probability of *Nlgn3*-deficient calyx synapses (Fig. 1), the *Nlgn3* deletion does not detectably change the organization of presynaptic RIM1 scaffolds.

However, the *Nlgn3* deletion caused a marked change in PSD-95–containing synaptic clusters and subsynaptic nanoclusters. While the total localization number (corresponding to the amount of protein) of PSD-95 was unaltered, the *Nlgn3* deletion induced a noticeable increase (~30%) in the size of PSD-95 synaptic clusters and a corresponding decrease (~30%) in the overall PSD-95 density (Fig. 3, I to M). This reduction in PSD-95 density was uniform and independent of the relative position of the PSD-95 nanoclusters within the synaptic cluster (Fig. 3K). At the subsynaptic level, the *Nlgn3* deletion did not change the number of PSD-95 nanoclusters per synapse (Fig. 3J) but greatly increased the volume of the nanoclusters (~45%) and decreased the PSD-95 density inside the nanoclusters correspondingly (~45%) (Fig. 3, N to P). The decrease in PSD-95 localization density was larger in the nanoclusters than in the synaptic cluster overall, which contributed to the reduction in the alignment of PSD-95 and RIM1 nanoclusters in *Nlgn3*-deficient calyx synapses (Fig. 2).

Together, these data demonstrate that the *Nlgn3* deletion induces a reorganization of postsynaptic specialization by increasing the size of the overall postsynaptic scaffold, expanding the volume of PSD-95–containing nanoclusters, and decreasing the density of PSD-95 molecules in synaptic clusters and subsynaptic nanoclusters. Note that an increase in the postsynaptic specializations was also observed with presynaptic deletions of RIM and RIM-binding proteins scaffolds at calyx synapses (*48*, *49*), suggesting that presynaptic signals organize postsynaptic scaffolds via an *Nlgn3*-dependent mechanism.

### The *Nlgn3* deletion alters GluA1 and GluA4 nanocluster organization

**Fig. 4.**
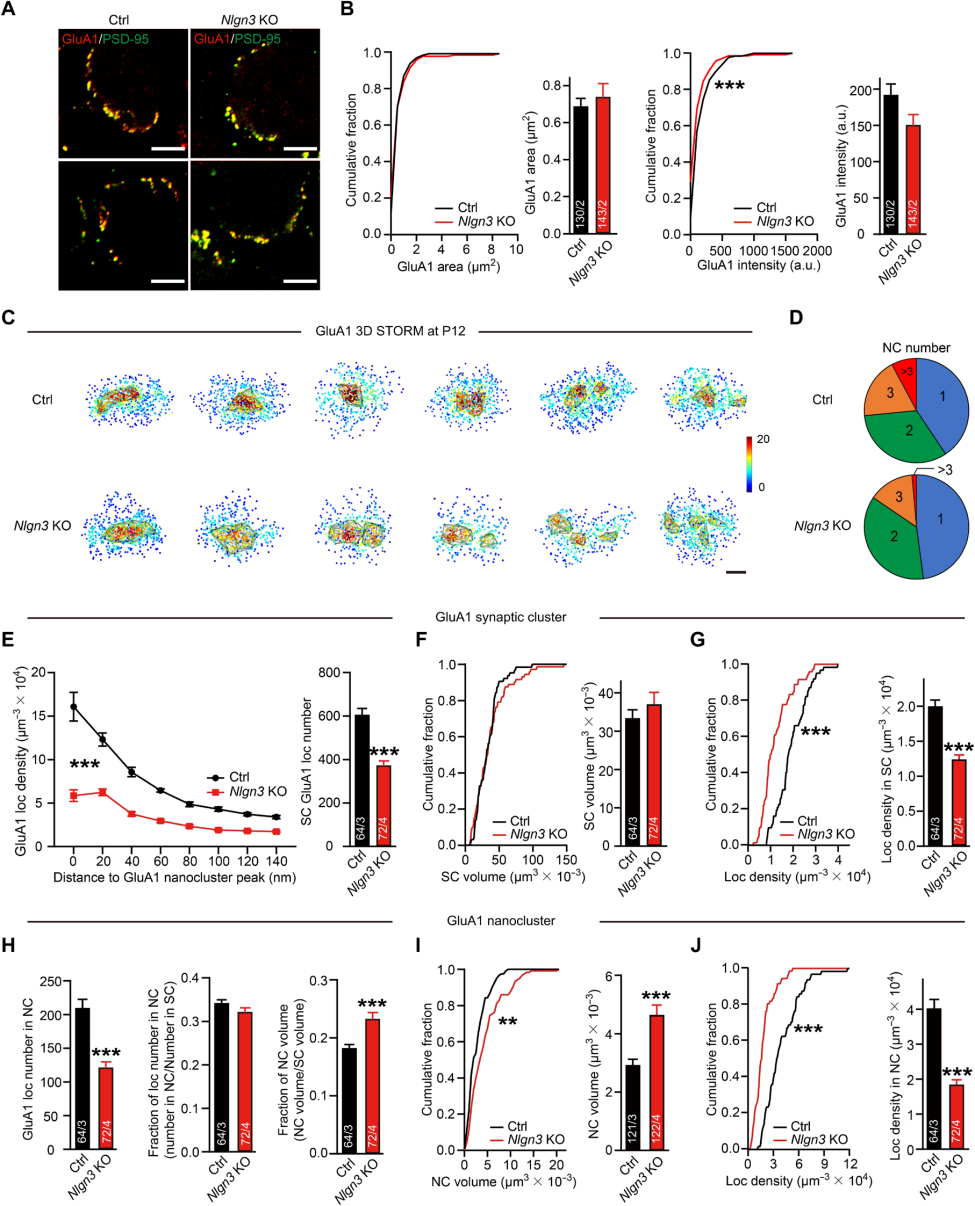
The *Nlgn3* deletion increases the size of GluA1 AMPAR nanoclusters but decreases the GluA1 AMPAR density in the calyx of Held synapses. Data were collected from mice at P12. (**A**) Example images of double staining of GluA1 (red) and PSD-95 (green) in P12 MNTB neurons of control (left) and *Nlgn3* KO (right) mice. (**B**) Analyses of GluA1 puncta area (left) and puncta intensity (right) in (A). (**C**) Example en face views of synaptic GluA1 local density maps in P12 MNTB neurons of control and *Nlgn3* KO mice. The gray lines depict the GluA1 nanocluster. Scale bar, 100 nm. (**D**) Pie charts showing the comparable percentage of the GluA1 nanocluster number per synapse at P12 in both groups. (**E**) Analyses of GluA1 local density as a function of the distance to the GluA1 nanocluster peak (left) and GluA1 local number within the synaptic cluster (right). (**F** and **G**) Analyses of GluA1 synaptic cluster volume (F) and local density in the synaptic cluster (G). (**H**) Analyses of GluA1 nanocluster. GluA1 local number (left), the fraction of GluA1 local number (middle), and the fraction of GluA1 nanocluster volume (right). (**I** and **J**) Analyses of GluA1 nanocluster volume (I) and local density in nanocluster (J). Data are means ± SEM. Numbers in bars represent the number of synapses/animals. Statistical significance was determined by a two-tailed Student’s *t* test (B and E to J), by the Kolmogorov-Smirnov test (B, F, G, I, and J), by the multiple unpaired *t* tests (E), or by the Mann-Whitney *U* test (D), with *P < 0.05, **P < 0.01, and ***P < 0.001.

AMPARs are thought to be anchored in synaptic specializations by binding to PSD-95 and its homologs via stargazins/transmembrane AMPA receptor regulatory proteins (TARPs) (*50*, *51*). The robust effect of the *Nlgn3* deletion on AMPAR-mediated transmission may be due to a change in the organization of AMPARs by PSD-95. At the calyx of Held synapse, fast-gating AMPAR isoforms are up-regulated developmentally (*15*, *52*). To investigate why the *Nlgn3* deletion impairs AMPAR-mediated EPSCs at the calyx of Held synapse, we used immunohistochemistry to image the organizations of GluA1 and GluA4 AMPARs at the calyx synapse, where they are the major AMPAR isoforms. Confocal microscopy of calyx sections stained for GluA1 revealed a similar synapse size in control and *Nlgn3* KO calyces but uncovered a small decrease in the overall GluA1 signal (~20%) (Fig. 4, A and B).

We next inquired whether the subsynaptic organization of GluA1 in calyx synapses is altered by the *Nlgn3* deletion. Consistent with the confocal results, the deletion of *Nlgn3* did not significantly change the size of the synaptic GluA1 clusters but caused a marked decrease in the overall localization number and density of GluA1 within the synaptic cluster (Fig. 4, E to G). When we analyzed the GluA1 nanoclusters, we found a similar number of nanoclusters per synapse (Fig. 4, C and D) in both groups. The *Nlgn3* deletion significantly reduced the number of GluA1 localizations within nanoclusters and increased the nanocluster volume (by ~40%), thereby producing a marked decrease in the GluA1 density within nanoclusters (~50%; Fig. 4J). The fraction of GluA1 localizations in nanoclusters was similar in both groups, but the relative volume of GluA1 nanoclusters within the synaptic cluster was significantly increased (Fig. 4, H and I). Together, these results reveal that the *Nlgn3* deletion causes a modest decrease in GluA1 receptors per synapse and a large increase in the size of GluA1 nanoclusters without affecting the number of the GluA1 nanoclusters.

**Fig. 5.**
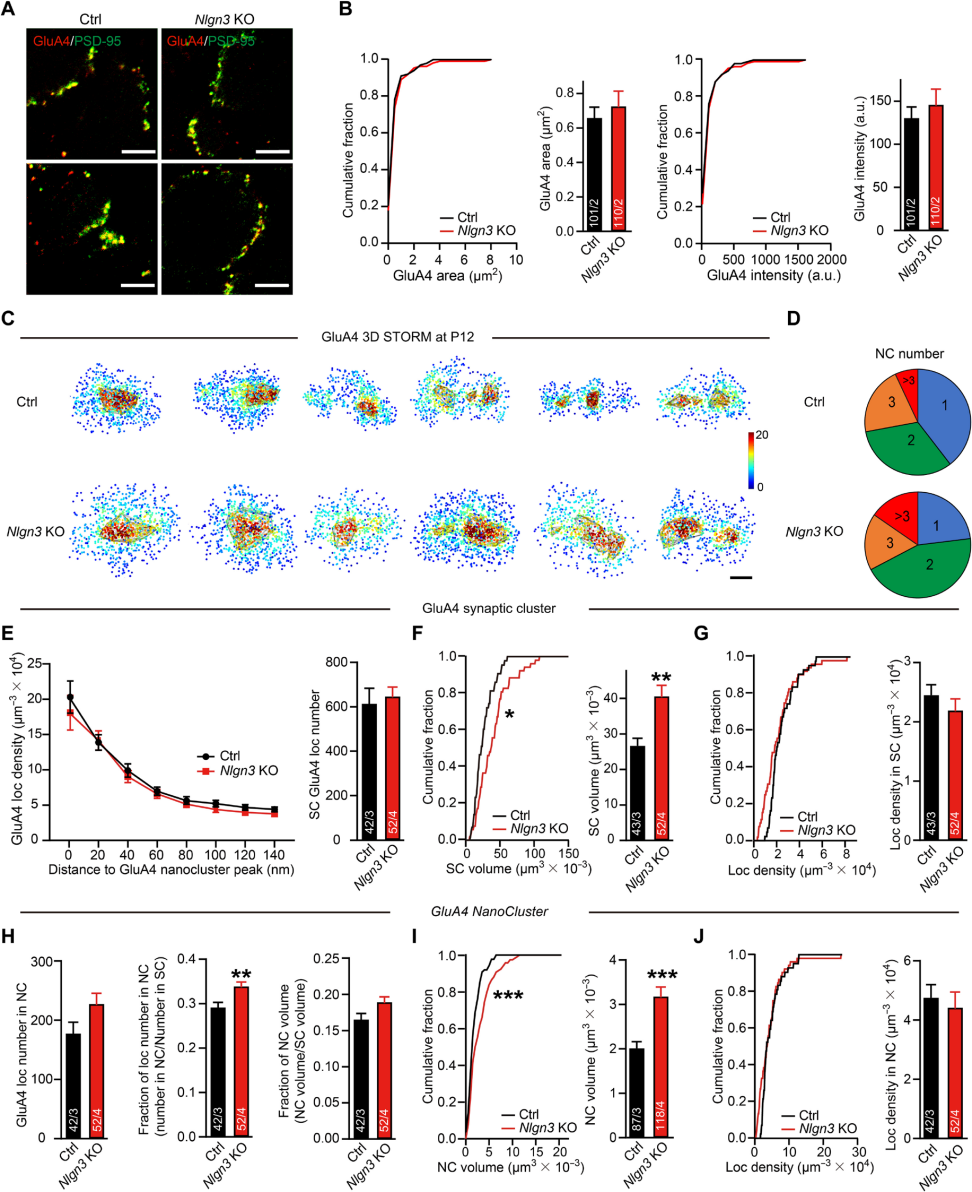
The *Nlgn3* deletion causes an increase in the size of GluA4 AMPAR nanoclusters similar to its effect on GluA1 AMPAR nanoclusters, but does not affect the GluA4 AMPAR density in the calyx of Held synapses. Data were collected from mice at P12. (**A**) Example images of double staining of GluA4 (red) and PSD-95 (green) in P12 MNTB neurons of control (left) and *Nlgn3* KO (right) mice. (**B**) Analyses of GluA4 puncta area (left) and puncta intensity (right) in (A). (**C**) Example en face views of synaptic GluA4 local density maps in P12 MNTB neurons of control and *Nlgn3* KO mice. The gray lines depict the GluA4 nanocluster. Scale bar, 100 nm. (**D**) Pie charts show a roughly similar percentage of the GluA4 nanocluster number per synapse at P12 in both groups. (**E**) Analyses of GluA4 local density as a function of the distance to the GluA4 nanocluster peak (left) and GluA4 local number within the synaptic cluster (right). (**F** and **G**) Analyses of GluA4 synaptic cluster volume (F) and local density in the synaptic cluster (G). (**H**) Analyses of GluA4 nanocluster. GluA4 local number (left), the fraction of GluA4 local number (middle), and the fraction of GluA4 nanocluster volume (right). (**I** and **J**) Analyses of GluA4 nanocluster volume (I) and local density in nanocluster (J). Data are means ± SEM. Numbers in bars represent the number of synapses/animals. Statistical significance was determined by a two-tailed Student’s *t* test (B and E to J), by the Kolmogorov-Smirnov test (B, F, G, I, and J), by the multiple unpaired *t* tests (E), or by the Mann-Whitney *U* test (D), with *P < 0.05, **P < 0.01, and ***P < 0.001.

For GluA4-containing receptors, the confocal imaging showed that the *Nlgn3* deletion did not affect the synapse size or protein amount, which is different from GluA1 (Fig. 5, A and B). STORM super-resolution imaging demonstrated that synaptic GluA4 clusters also contained nanoclusters (Fig. 5C). The *Nlgn3* deletion increased the synaptic cluster volume of GluA4 while having no significant effects on the overall localization number or localization density within synaptic clusters (Fig. 5, E and G). At the subsynaptic level, similar to GluA1, the *Nlgn3* deletion had no significant effects on the number of GluA4 nanoclusters per synapse (Fig. 5, C and D) and greatly expanded the volume of GluA4 nanoclusters (by ~40%). However, *Nlgn3*-deficient synapses showed no significant changes in the GluA4 localization number and density within nanoclusters (Fig. 5, H and J) and had a slight increase in the fraction of GluA4 localizations in nanoclusters (Fig. 5I). Therefore, while the *Nlgn3* deletion increased the nanocluster volume for both GluA1 and GluA4, these two receptors had distinct patterns in the modulation of their synaptic organizations by *Nlgn3*.

Together, the STORM analysis reveals that PSD-95, GluA1, and GluA4 are organized into similar nanoclusters in the calyx of Held synapses and that the Nlgn3 deletion does not impair the number of nanoclusters but produces a large increase in the volume of these nanoclusters. In addition, the *Nlgn3* deletion induces an increase in the overall synaptic cluster volume as visualized using GluA4 and PSD-95 staining, and a modest decrease in GluA1 and PSD-95 but not GluA4 content.

### Computational modeling indicates that AMPAR nanocluster disorganization impairs high-fidelity synaptic transmission

**Fig. 6.**
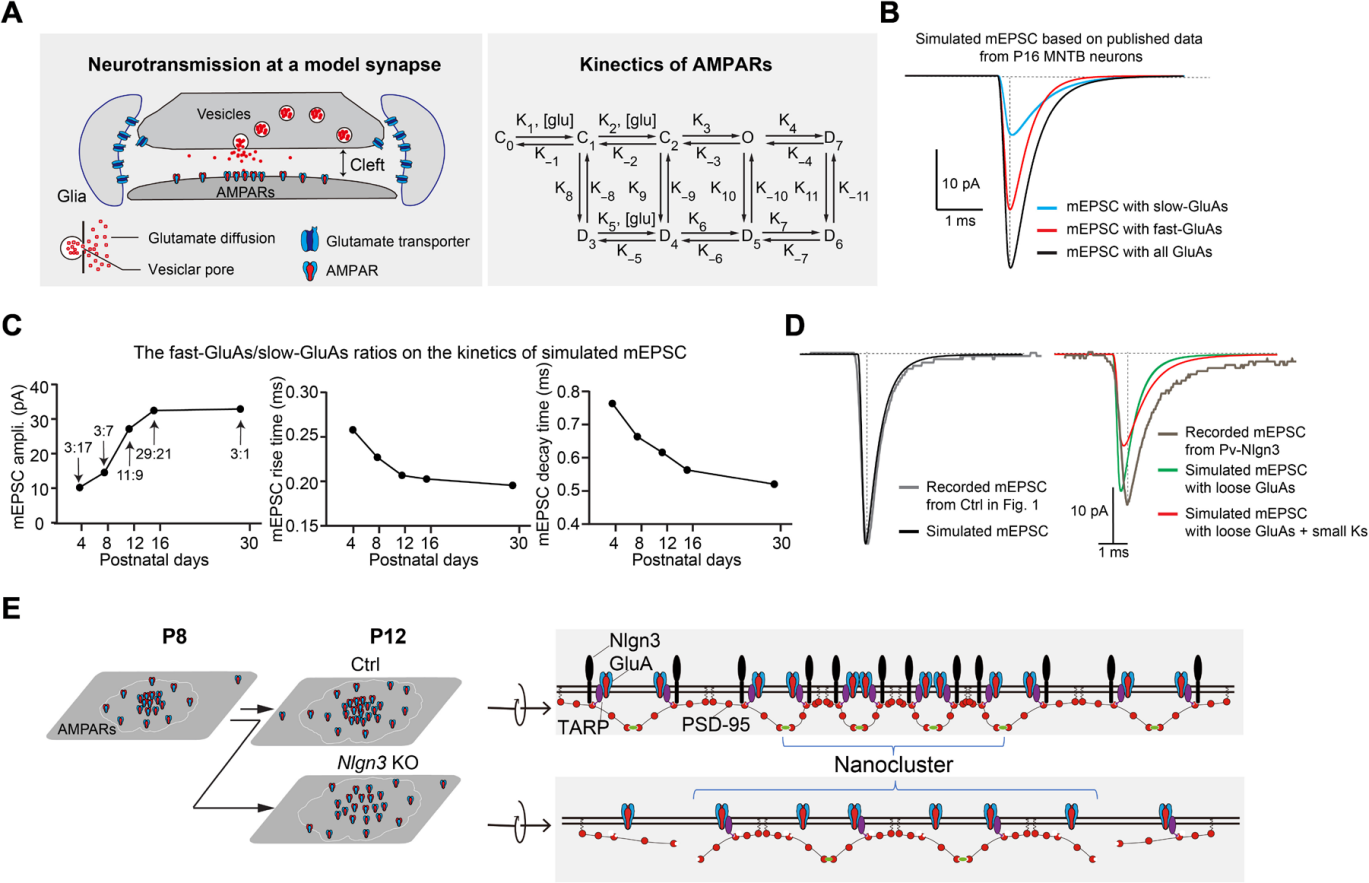
Modeling indicates that the dilution of GluAs into larger nanoclusters accounts for the decrease in synaptic strength in the calyx of Held synapses. (**A**) 3D synaptic geometry used in the simulations of mEPSC at the calyx of Held with fast-gating and slow-gating GluAs. The vesicle was placed at different locations over the central PSD. The right panel shows AMPAR kinetic model. (**B**) Simulated slow-gating GluAs–mediated (blue), fast-gating GluAs–mediated (red), and combined (black) mEPSC from published P16 MNTB neurons. Each trace is an average of 160 individual randomizations. (**C**) Plots of amplitude, rise time, and decay time of simulated mEPSC at different developmental stages. According to previous reports (*15*, *54*), the ratio of fast-GluAs:slow-GluAs used is 3:17, 3:7, 11:9, 29:21, and 3:1 for P4, P8, P12, P16, and P30 MNTB neurons, respectively. (**D**) Recorded and simulated P12 mEPSCs from MNTB neurons in Ctrl mice (left). Recorded and simulated P12 mEPSCs from MNTB neurons in Pv-Nlgn3 mice (right). GluA parameters were obtained in super-resolution studies based on PSD-95 density (Fig. 2F). Diffused GluAs alone do not result in slower kinetics (green trace), while reducing the kinetics of GluA activation by multiplying a factor (0.6) simulated the mEPSC of P12 *Nlgn3* KO MNTB neurons well (red trace). (**E**) Working model of the effect of the *Nlgn3* deletion on GluA clustering at the calyx of Held. *Nlgn3* binds to PSD-95 scaffolds that, in turn, bind to GluAs, thereby inducing their clustering. Loss of *Nlgn3* enables PSD-95 to diffuse out of the tight nanocluster that is aligned with presynaptic clusters, thereby resulting in looser clustering of PSD-95 and of GluAs that bind to PSD-95 via stargazins/TARPs.

While the specific modulation of *Nlgn3* on organizations of different proteins requires further investigation, it is critical to figure out whether the observed structural changes could account for the functional changes that we recorded at the calyx of Held synapse. We therefore adopted an alternative method to address this issue—building and testing computational models of AMPAR-mediated synaptic transmission at the calyx of Held. To build our model, we assumed that spontaneous vesicle fusions that mediate mEPSCs happen randomly over the RIM synaptic clusters, and that action potential–triggered vesicle fusions are localized to RIM nanoclusters (tables S1 to S4) (*8*). The released glutamate from presynaptic vesicles then activates postsynaptic AMPARs via a process containing nine kinetic steps (Fig. 6A) (*53*). The sizes of the active zone and PSD-95 were set on the basis of our STORM data (Figs. 2F, 4, and 5) and electron microscopic (EM) data collected from mice at similar development ages (*54*). Since both fast- and slow-GluAs contribute to synaptic transmission at the calyx of Held (*15*, *52*), we included their contributions in our model (table S2).

To test the robustness of our model, we first simulated the mEPSCs from P16 to P18 MNTB neurons of both GluA4 KO and littermate control mice reported in a previous study (*15*, *52*). We obtained a satisfying fit of the mEPSCs from both genotypes, with fast-GluA4 contributing ~65% to the amplitude of the mEPSCs with much faster kinetics (Fig. 6B). We then applied the different ratios of fast- versus slow-gating GluAs that were determined in previous studies (table S2) to our model. This resulted in a predicted reduced decay time of mEPSCs during development (Fig. 6C and table S2), mimicking the profiles of mEPSC decay times during development described earlier (*54*). Thus, those two tests suggest that our model robustly simulates mEPSCs at the calyx of Held synapse.

We then directly tested whether the changes in the subsynaptic organization of GluAs that we observed in Nlgn3 KO mice could account for the resulting functional changes in mEPSCs. We first simulated the mEPSCs recorded at control calyx of Held synapses from P12/13 wild-type mice (Figs. 1 and 6D, right) and then applied the changes in the GluA1 and GluA4 distribution that we observed by STORM to our model. This simulation suggested that the changes in GluA distribution produced a decrease in mEPSC amplitude without a major alteration of the kinetics (fig. S2B). To include the potential contribution of the misalignment of pre- and postsynaptic elements on the mEPSCs, we then redistributed the GluAs as the normalized pattern of PSD-95 in Fig. 2, which resulted in a better fit of the mEPSC amplitude but again did not predict changes in kinetics (Fig. 6D, green, and table S3). Therefore, our model confirmed that the observed structural changes contribute to the smaller amplitude of mEPSC in Nlgn3 KO mice (Fig. 1) but did not predict the slower kinetics of mEPSCs (Fig. 6D, green). We also systematically analyzed the parameters that affect glutamate dynamics in the synaptic cleft on the mEPSC kinetics and did not get a better fitting of kinetics (fig. S2, C to F). However, when we reduced the rate constants of transitions between different receptor states (Ks) in our GluA activation model, we were able to decrease the kinetics of mEPSCs (Fig. 6D, red, and fig. S2G), mimicking the slower kinetics of mEPSCs from P12 *Nlgn3* KO calyx of Held synapses (Fig. 1, E and G). Moreover, the slower kinetics with smaller Ks further reduced the mEPSC amplitude (~50%), implying that other mechanisms might contribute to the slower kinetics after the *Nlgn3* deletion (Fig. 1). Since the phenotype of the evoked EPSCs phenotype is bigger than that of the mEPSCs in *Nlgn3* KO mice (Fig. 1), we further tested the impact of GluA changes on the amplitude of evoked EPSCs in our model. Similarly, we found that the impact of the redistribution of GluAs was robust in the simulations of the amplitude of evoked EPSCs (fig. S3), consistent with what we found in our electrophysiological recordings (Fig. 1, B and C).

## DISCUSSION

Here, we examined how Nlgn3, a postsynaptic adhesion molecule that binds to presynaptic neurexins, controls the nanoscale organization of a central synapse, and how the synaptic organizer function of Nlgn3 optimizes synaptic transmission. We addressed these questions at the calyx of Held synapse, a paradigmatic brainstem synapse involved in sound localization that allows analyses of synaptic properties at high resolution. We found that deletion of *Nlgn3* reduced the strength of excitatory calyx synapses and that this reduction was due to a markedly selective impairment in the nanoscale arrangement of AMPARs at calyx synapses without disrupting overall synapse formation. Specifically, the *Nlgn3* deletion had no effect on the organization of presynaptic clusters of RIM1 or the assembly of RIM1 into nanoclusters but increased the volume of postsynaptic PSD-95–containing overall synaptic clusters and PSD-95 nanoclusters quite markedly (30 to 50% increase; Fig. 3). Moreover, the *Nlgn3* deletion greatly expanded the volume of GluA1 and GluA4 AMPAR nanoclusters (50 to 60% increase; Figs. 4 and 5) but decreased the density of these molecules in the synaptic cluster and nanoclusters. Since PSD-95 is a subsynaptic scaffolding protein that binds to Nlgn3 protein and that anchors AMPARs in postsynaptic specializations, these data suggested that the loss of *Nlgn3* causes an expansion of PSD-95– and AMPAR-containing nanoclusters with lower concentrations of AMPARs. We incorporated these nanostructural changes into a numerical model of synapses, which predicted that the nanoscale organization changes would reduce quantal synaptic transmission (Fig. 6), as observed experimentally (Fig. 1). Therefore, our results uncover an essential role for Nlgn3 in controlling the nanoarchitecture of a central excitatory synapse in vivo.

Our data suggest that *Nlgn3* enables the recruitment of postsynaptic AMPARs into tight nanoclusters directly opposing RIM1 nanoclusters in the presynaptic active zone that corresponds to neurotransmitter release sites. Synaptic transmission and plasticity are shaped by the dynamic organization of key effector molecules within pre- and postsynaptic compartments (*55*). Presynaptically, voltage-gated calcium channels at the calyx of Held undergo a transformation from microdomain coupling at immature (<P10) calyx synapses to nanodomain coupling at mature (>P15) synapses (*56*–*58*). Postsynaptically, the glutamate receptor composition at the calyx synapse changes during the same developmental period, rendering AMPAR-EPSCs faster and reducing the NMDAR-EPSC amplitude as the synapse matures (*59*, *60*). The clustering of GluA1 and GluA4 at P12 in the current study likely represents an intermediate stage during the maturation of pre- and postsynaptic specializations at the calyx synapses. This maturation requires Nlgn3, and deletion of *Nlgn3* causes a partial uncoupling of pre- and postsynaptic specializations, leading to the disorganization of AMPAR nanoclusters and impairments in synapse function.

We found that *Nlgn3* is indispensable for the maintenance of PSD-95 and GluA1 and GluA4 nanoclusters but is also required for synaptic transmission as such, which is consistent with earlier results (*30*, *33*, *61*). The *Nlgn3* deletion causes an expansion of synaptic clusters and nanoclusters containing PSD-95 and AMPARs but does not abolish these clusters. Since different PDZ domains of PSD-95 bind to Nlgns (*62*), and to stargazins/TARPs (*63*), PSD-95 is in a great position to coordinate trans-synaptic neurexin-Nlgn adhesion interactions with postsynaptic recruitment of AMPARs (*51*, *64*). As a result, more diffusive PSD-95 after the *Nlgn3* deletion could directly result in a loose nanocluster of GluA1 and GluA4. PSD-like assemblies (containing glutamate receptors) could be reconstituted in vitro with major excitatory PSD scaffold proteins (*65*). Therefore, it is likely that *Nlgn3* maintains the content and clustering of AMPARs at the calyx of Held by assembling Nlgn3/PSD-95/GluA1 and Nlgn3/PSD-95/GluA4 complexes into tight nanoclusters.

Our study raises multiple new questions. Since *Nlgn3* modulates the synaptic function at both the excitatory and inhibitory synapses (*27*, *30*, *33*, *61*, *66*–*68*), the organization of AMPAR nanoclusters is clearly not its only function. It is conceivable that at inhibitory synapses a similar role may pertain, but the molecules involved are unclear. Moreover, it is not certain that Nlgn3 is regulated by presynaptic neurexins in the function we describe here, since other ligands could be important. Overexpressing presynaptic β-neurexins in human embryonic kidney (HEK) 293 cells could induce postsynaptic PSD-95 accumulations in cocultured neurons, which in turn recruited NMDARs and AMPARs in an activity-dependent manner (*64*). These data support the notion that presynaptic neurexins modulate the postsynaptic PSD-95/AMPAR/NMDAR complex. It is interesting that the deletions of *Nlgn1* and *Nlgn3* have distinct effects on synaptic transmission at the calyx and hippocampal synapses, suggesting that these Nlgn isoforms, their high degree of similarity notwithstanding, perform different functions. Moreover, Leucine Rich Repeat Transmembrane proteins (LRRTMs) and cerebellins, which are other families of neurexin ligands, were previously shown to regulate AMPARs (*7*, *69*), and their relation to Nlgns remains unstudied.

Our computational model confirmed that the reorganization of postsynaptic AMPARs regulates synaptic strength. This model also predicted that regulating the rate constants of transitions between different receptor states in GluAs changes the kinetics of mEPSCs. The smaller Ks used in the simulation in *Nlgn3* KO synapses suggests a slower activation of the GluA channel, which may arise from changes in stargazins/TARPs (*70*) and/or other modulators that bind to Nlgn3/PSD-95. While this model could not perfectly simulate the amplitude, rise, and decay time of mEPSCs at the same time shown in *Nlgn3* KO synapses (Fig. 1, F, and G), some other enigmatic factors contribute to the modulation. What is more, the model assumed that the release sites for mEPSC and EPSC are different, consistent with recent studies that spontaneous and evoked neurotransmission comprises independent neuronal signal transduction pathways that may operate in a spatially segregated manner (*71*). Our model also predicted that this difference in the modulation of EPSC and mEPSC amplitudes depended on the amount of released glutamate. A larger number of transmitters (strong synapse with more-loaded transmitters) reduced the difference between the impacts of GluA nano-organization on the EPSC and mEPSC, while this difference was stronger for weaker synapses with less-loaded vesicle, which may account for the higher sensitivity of EPSCs to GluA clustering than mEPSCs in the hippocampus (*7*, *44*). The initial model that we adopted compared different AMPAR schemes; on this basis, we changed the algorithm to satisfy the idea that we can adjust the ratio and distribution of fast-GluAs and slow-GluAs. To the best of our knowledge, we are the first to incorporate the STORM-based GluA distribution into a synaptic transmission model. Different glutamatergic synapses might use different combinations of fast- and slow-GluAs for their synaptic transmission; our current model is the first computational study with consideration of different GluAs on synaptic transmission. It is crucial to consider the contribution of different GluAs in any further modeling study.

In summary, our results establish a discrete but important role for *Nlgn3* at central calyx synapses in organizing AMPAR nanoclusters, thereby enabling fast and reliable synaptic transmission. These results assign a defined function to Nlgn3 that is important for optimizing synaptic transmission, but that—consistent with previous results (*30*, *33*, *34*, *61*, *67*)—is not in itself required for excitatory synapse assembly. Moreover, these results suggest a potential rationale for the association of *NLGN3* mutations with autism in human patients, given that these mutations cause a marked impairment in the behavioral properties of the patients but do not in themselves completely incapacitate these patients.

## MATERIALS AND METHODS

### Mouse husbandry

The following mouse lines were used for breeding and the experiments: Pv-Cre (*33*) and *Nlgn1* cKO, and *Nlgn3* cKO (*68*). Briefly, we used our previously generated Nlgn1 cKO mice in which exon 7 is deleted by Cre-recombinase (*68*) and the Nlgn3 cKO mice in which the start codon is deleted by Cre-recombinase (*61*). The Pv-Cre mice that we used express Cre-recombinase being driven by the Pv promoter (*72*). All analyses were performed on littermate mice for physiology (P8 to P13). The in vivo stereotactic injections were carried out at P0. All analyses were performed on mice whose genotype was blinded to the experimenter. All procedures conformed to Guidelines for the Care and Use of Laboratory Animals and were approved by the Peking University Shenzhen Graduate School on Laboratory Animal Care and the Stanford University Administrative Panel on Laboratory Animal Care.

### Virus generation and in vivo stereotactic injection

Lentiviruses with vector target construct [synapsin-Cre-EGFP (enhanced GFP) and synapsin-EGFP] were prepared as described (*61*). Intracranial injection of the virus in vivo was performed using a stereotaxic instrument (David Kopf) under 2 min of ice anesthesia of P0 pups. A small volume (∼1 μl) of concentrated virus solution (titer of the virus) was injected into MNTB at a speed of 0.5 μl/min using a syringe pump (Harvard Apparatus). The injection needle was withdrawn 30 s after the end of the infusion. Electrophysiology studies were performed between P8 and P9 after the P0 injection. Only the coronal slices confirmed with infected MNTB neurons were selected for use. We injected 1 μl of lentivirus solution into MNTB to ensure quantitative infiltration of the nucleus from both sides. The virus-mediated Cre expression fused with GFP in the MNTB after 8 to 9 days of expression showed a preferential neuronal morphology. Hence, virus injection into MNTB at P0 allowed us a selective expression of Cre-recombinase in MNTB neurons.

### Electrophysiology

Transverse 200-μm-thick slices of the brainstem containing the MNTB were made according to standard procedures with a vibratome (Leica VT1200S), using mutant and control mice at the age between P11 and P13, similar as described previously (*33*). The extracellular recording solutions contained 125 mM NaCl, 25 mM NaHCO_3_, 2.5 mM KCl, 1.25 mM NaH_2_PO_4_, 25 mM glucose, 0.4 mM ascorbic acid, 3 mM myo-inositol, 2 mM Na-pyruvate, 2 mM CaCl_2_, and 1 mM MgCl_2_ (pH 7.4, when bubbled with 95% O_2_/5% CO_2_). For the recordings of fiber stimulation–evoked EPSCs, picrotoxin (50 μM) and strychnine (2 μM) were added to the extracellular solution. For recordings of mEPSCs in MNTB neurons, tetrodotoxin (1 μM), picrotoxin (50 μM), strychnine (2 μM), and d,l-2-amino-5-phosphonovaleric acid (APV) (50 μM) were added. We examined and analyzed the mEPSC after confirming calyceal EPSC in each recording. For recordings of AMPAR-mediated EPSCs, APV (50 μM), picrotoxin (50 μM), and strychnine (2 μM) were added. Internal solutions in pipette included 140 mM Cs-gluconate, 20 mM tetraethylammonium, 10 mM Hepes, 5 mM Na_2_-phosphocreatine, 4 mM MgATP, 0.3 mM Na_2_GTP, 5 mM Cs-EGTA, and 0.2 mM spermine (pH 7.2). Whole-cell recordings in the voltage-clamp mode were made with an Axon amplifier, under visualization of neurons with an upright microscope (BX51WI, Olympus, Tokyo, Japan) equipped with a 40× water immersion objective (Zeiss). Postsynaptic patch pipettes had resistances of 2 to 3 megohms, and series resistance (4 to 7 megohms) was compensated between 75 and 85%. The residual Rs errors in postsynaptic recordings were comparable in both genotypes (1 ± 0.1 megohms) and were not corrected off-line. Cells were held at −60 mV in voltage clamp; membrane potentials were not corrected for liquid junction potentials.

### Confocal imaging

Mice of age P12 were anesthetized, and brain tissues containing MNTB were dissected out and frozen in dry ice immediately. Tissue slices were sectioned on cryostat sections (8 μm thickness) and collected on #1.5 glass coverslips. The sections were fixed with 4% paraformaldehyde (PFA) for 10 min and washed with phosphate-buffered saline (PBS) plus 100 mM glycine. Then, the sections were permeabilized and blocked [5% bovine serum albumin (BSA) and 10% donkey serum in 0.3% Triton X-100] at room temperature, followed by incubation overnight at 4°C with primary antibodies (rabbit anti-GluA1, Merck Millipore, ab1504, 1:400; rabbit anti-GluA4, Merck Millipore, ab1508, 1:400; mouse anti–PSD-95, NeuroMab, 75-028, 1:200; rabbit anti-RIM 1/2, Synaptic Systems, 140203, 1:500). Secondary antibodies were then applied for 1 hour at room temperature. Alexa Fluor 647–conjugated donkey anti-rabbit secondary antibodies (Jackson ImmunoResearch, 711-605-152) were used for GluA1 or GluA4. CyTM3B-conjugated (Mono NHS Ester, 16889934) donkey anti-mouse secondary antibodies were used for PSD-95. Atto 488–conjugated donkey anti–guinea pig secondary antibodies (Jackson ImmunoResearch, 715-545-151) were used for vGluT1.

Imaging was carried out on a Nikon ECLIPSE Ti2 inverted microscope equipped with a perfect focusing system and a 100×/1.45 oil immersion objective controlled with NIS-Elements AR 4.30.02 software. All confocal data were analyzed using Fiji imaging analysis software (http://fiji.sc/Fiji).

### Simulations

Quantal release of glutamate was carried out with MATLAB (version R2021a; MathWorks, Natick, MA, USA) by using a Monte Carlo algorithm that simulated the stochastic behavior of molecule diffusion and dynamic binding to AMPARs in a complex microenvironment with a time step of 0.5 μs.

#### Simulation of AMPAR distribution

Considering its unique structure, the calyx of Held could be considered as a large parallel arrangement of a few hundred active zones aligned to the corresponding postsynaptic sites (*73*). Thus, the extracellular space between the presynaptic and postsynaptic membrane was regarded as two paralleled coaxial cylinders of 0.5-μm length, with a 28-nm synaptic cleft between the cylinders (*9*).

We adopted a previous model to describe the number of different internal states of AMPARs (Fig. 6A) (*53*). Since both slow- and fast-GluAs contribute to synaptic transmission at the calyx of Held, we include both fast- and slow-gating GluAs in our model. The radiuses of nano- and synaptic clusters of the active zone and PSD-95 were calculated on the basis of previous EM data (*73*) and our STORM data. A total of 100 fast- and slow-gating GluAs were placed as assigned with the ratio of the different development periods (table S2) (*15*, *52*). Owing to the distinct deviations in *x*/*y* and *z* (see details in the “3D STORM imaging” section), we could not get the radius from STORM directly. Thus, the radiuses of synaptic clusters and nanoclusters (table S2) were estimated by the radius of synaptic clusters obtained from the ultrastructure of P14 MNTB neurons (*73*) and our STORM data of GluA1/A4 in P12 MNTB neurons.

Considering the potential contribution of the misalignment on mEPSC, the distribution of both fast- and slow-gating GluAs in the current simulation was further estimated on the basis of the PSD-95 localization density, which was aligned to the presynaptic RIM1, and subdivided into three subregions: 0 to 60 nm, 60 to 100 nm, and 100 to 140 nm (Fig. 2F and table S3). The PSD-95 density at 140 nm is normalized to 0.5 (the dashed line in Fig. 2F) to get a threefold density in the nanocluster (<60 nm) than outside (60 to 140 nm) in the control group, as shown in the heat map (Fig. 2E). In each subregion, GluAs are uniformly distributed with a ratio of slow/fast-GluAs = 0.45/0.55 for P12 MNTB neurons. The detailed number of GluAs in each region is listed in table S3. We defined the radius of release sites constrained to the PSD-95 synaptic cluster to simulate mEPSC. For EPSC, we constrained release sites regarding the PSD-95 nanocluster. Cluster GluAs are subdivided into three regions based on the PSD-95 density as described above. Random GluAs are distributed uniformly throughout the PSD-95 synaptic cluster.

#### Simulation of glutamate release and AMPAR activation

The initial fusion pore conductance of a single vesicle at the calyx of Held is >375 pS (*74*), and the relationship between transmitter flux and conductance (*75*) permits a calculation of vesicular expansion time (τ = 73 γ^−1^ μs, where γ is the fusion pore conductance in nS) of 0.2 ms to release its all transmitters. The number of glutamate molecules in the vesicle was set to 8000 at P12. After releasing, glutamate molecules do Brownian motion at a diffusion rate of 0.4 μm^2^/ms.

When glutamate hits the membrane or even AMPAR, it will not be bound but reflected. A nine-state AMPAR reaction scheme was taken from a previous study (*53*). An AMPAR was run against the glutamate transients to calculate the open probability of individual AMPAR. Every AMPAR was regarded as a circular area with a radius of 10 nm and its internal state depending on the number of glutamates hitting this area during a one-time step. The effect of glutamate binding to GluAs is negligible for the much greater number of glutamates (8000) than GluAs (100). The rate constants of slow-GluAs and fast-GluAs were initially set as previous study (*53*) and were adjusted within the constraint of microscopic reversibility (*76*). Transporters were uniformly distributed on the glial sheath, which surrounded the synapse (fig. S2A). The default density of transporters was 5000/μm^2^ for P12 neurons, and the distance between synaptic edge and glia was 40 nm (*9*).

The traces shown here were mean values of 160 runs with release sites randomly distributed through the active zone with a radius equal to synaptic cluster for mEPSC and nanocluster for EPSC. All the default parameters we used are listed in tables S1 to S4 unless stated. The 10 to 90% rise time and decay time were calculated by fitting the rise and decay phases of mEPSCs with double exponential functions. EPSC at time *t* is generated by

$$I(t)=[g\times n(t)]\times (V_{m}-V_{\text{GluAs}})$$ (1)

where *g* is the single-channel conductance set at 31 pS for slow-GluAs and 45 pS for fast-GluAs (*13*), *n*(*t*) is the number of open GluAs at time *t*, *V*_m_ is the resting membrane potential, and *V*_GluAs_ is the reversal potential of GluAs.

### 3D STORM imaging

For STORM imaging, immunohistochemistry was performed following protocol as described previously (*8*, *45*). Mice of age P12 were annuitized, and brain tissues containing MNTB were dissected out and frozen in dry ice immediately. Tissue slices were sectioned on cryostat sections (8 μm thickness) and collected on #1.5 glass coverslips. The sections were fixed with 4% PFA for 10 min and washed with PBS plus 100 mM glycine. Then, the sections were permeabilized and blocked (5% BSA and 10% donkey serum in 0.3% Triton X-100) at room temperature, followed by incubation overnight at 4°C with primary antibodies (rabbit anti-GluA1, Merck Millipore, ab1504, 1:400; rabbit anti-GluA4, Merck Millipore, ab1508, 1:400; mouse anti-PSD-95, NeuroMab, 75-028, 1:200). Secondary antibodies were then applied for 1 hour at room temperature. Alexa Fluor 647–conjugated donkey anti-rabbit secondary antibodies (Jackson ImmunoResearch, 711-605-152) were used for GluA1. Cy3B-conjugated (Mono NHS Ester, 16889934) donkey anti-mouse secondary antibodies were used for PSD-95.

Imaging was carried out on a Nikon ECLIPSE Ti2 inverted microscope equipped with a perfect focusing system and a 100×/1.49 total internal reflection fluorescence oil-immersion objective controlled with NIS-Elements AR 4.30.02 software. The typical incident power was ~40 mW (measured through the objective). MNTB tissue slices were imaged in a freshly made STORM imaging buffer containing 50 mM tris, 10 mM NaCl, 20% glucose, glucose oxidase (56 μg/ml) (Sigma-Aldrich), catalase (18 μg/ml) (Sigma-Aldrich), and 100 mM cysteamine (Sigma-Aldrich). To reduce background fluorescence while maximizing the depth of view, we adjusted the incident angle of the excitation beam to near but less than the critical angle to achieve oblique illumination of the sample. Emission was collected with a complementary metal-oxide semiconductor (CMOS) camera (ORCA-Flash4.0, Hamamatsu) at a frame rate of 50 Hz and stored as images with a pixel size of 160 nm. A total of 50k images were collected for each channel. TetraSpeck beads (100 nm; Invitrogen) deposited on a coverslip were localized for generating the calibration curves. In our system, the average deviation of the bead localizations after the correction was 10 to 15 nm in *x*/*y* and 40 to 50 nm in *z*. Localization detection, calibration, and drift correction were done using the NIS-Elements AR analysis 4.40.00 software. Localization coordinates were then rendered into images (pixel size of 5 nm) using a 2D Gaussian kernel (σ = 15 nm) with custom routines in MATLAB (MathWorks).

Detailed analyses on synaptic clusters were performed using custom routines in MATLAB as described previously (*8*, *47*). A synaptic cluster (equivalent to a PSD) was identified in a 2D scatter plot of all localizations. By rotating a 3D scatterplot of localizations of a selected potential synaptic cluster, we evaluated the data quality and selected only those with clear edges (e.g., no nearby third cluster that may indicate more than two synaptic clusters nearby) for further analysis. To define a synaptic cluster on the random background, the nearest neighbor distances (NNDs) between localizations were calculated and the mean + 2 × SD of NND was used as a cutoff to divide the localizations into subclusters. All localizations outside the primary subclusters were considered to be a background and were not used in further analysis. The border of the synaptic cluster was defined using the alpha shape of the set of 3D localizations with α = 150 nm.

Nanoclusters were detected on the basis of local density, which was defined as the number of molecules within a radius of 2.5 times the standard median NDD for the calculation of the density of the synaptic cluster (*47*). The threshold of local density for nanocluster detection was defined as mean (LD0) + 4 × std (LD0), where LD0 is the local density of a randomized cluster with the same overall density as the synaptic cluster. All localizations with a local density larger than this threshold were considered within nanoclusters. These localizations were then divided into subclusters with a “top-down” divisive strategy with a minimal peak-to-peak distance of 80 nm. Last, to be counted as nanoclusters, those subclusters had to meet a set of criteria, including the number of localizations (≥8 localizations), which were derived empirically based on tests on our measured and simulated synapses to reduce the false positives arising from repeated localizations of the same molecule.

### Data analysis

Data analysis was performed using the IgorPro and GraphPad Prism 9.0.0 software. Data are reported as average ± SEM values, and statistical significance was evaluated using unpaired, two-tailed Student’s *t* test, multiple unpaired *t* tests, Mann-Whitney *U* test, single-factor analysis of variance (ANOVA), and Kolmogorov-Smirnov test for cumulative curves. Statistical significance was accepted at *P* < 0.05. Asterisks above brackets in data bar graphs indicate the level of statistical significance (*P < 0.05, **P < 0.01, and ***P < 0.001).

## Supplementary Materials

### This PDF file includes:

Figs. S1 to S3 Tables S1 to S4 References

## Notes

View/request a protocol for this paper from *Bio-protocol*.
