# Supplementary material for: Neuroligin-3 confines AMPA receptors into nanoclusters, thereby controlling synaptic strength at the calyx of Held synapses
Source: Sci Adv. 2022 Jun 15;8(24):eabo4173. doi: 10.1126/sciadv.abo4173 (PMC9200272; doi:10.1126/sciadv.abo4173)
Supplement: Supplementary file 1 — Figs. S1 to S3 Tables S1 to S4 References [file sciadv.abo4173_sm.pdf]

Science Advances
AAAS

Supplementary Materials for

Neuroligin-3 confines AMPA receptors into nanoclusters, thereby controlling synaptic strength at the calyx of Held synapses

Ying Han *et al.*

Corresponding author: Bo Zhang, zbo@pku.edu.cn; Thomas C. Südhof, tcs1@stanford.edu; Ai-Hui Tang, tangah@ustc.edu.cn

Sci. Adv. **8**, eabo4173 (2022)
DOI: 10.1126/sciadv.abo4173

This PDF file includes:

Figs. S1 to S3
Tables S1 to S4
References

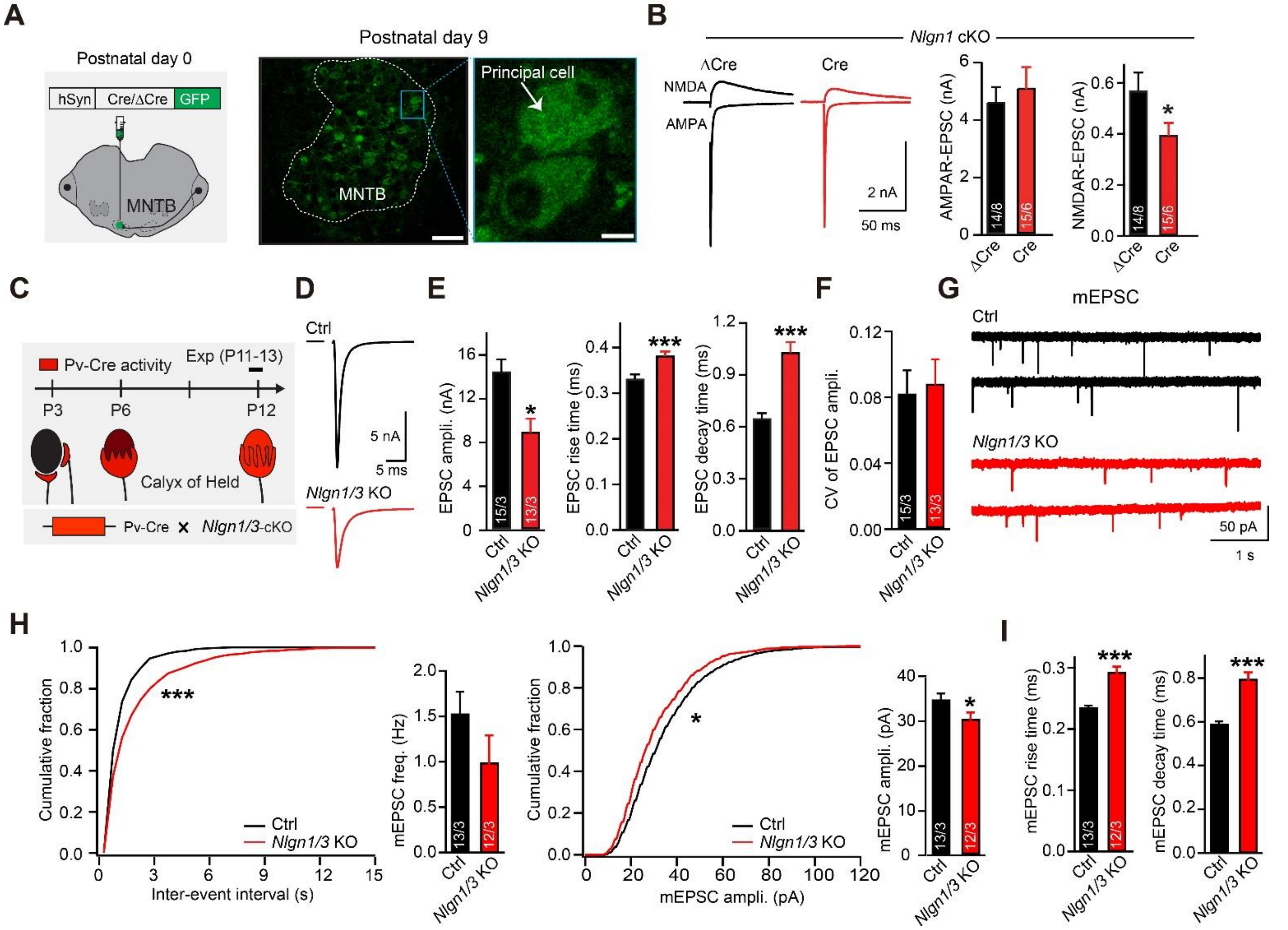

fig. S1.

Nlgn3, but not Nlgn1, regulates AMPAR-mediated EPSCs at the calyx of Held.

(**A**), Experimental strategy. A stereotactic injection approach and representative fluorescence images of MNTB principal neurons in brainstem slices cut at P9 from a mouse that had been stereotactically injected at P0 with lentiviruses expressing eGFP-tagged Cre or control virus expressing eGFP. Calibration bar: 25 µm (left) and 5 µm (right). (**B**), Virally induced ablation of *Nlgn1* in MNTB neurons reduces NMDAR-mediated EPSCs but has no effects on AMPAR-mediated EPSCs at the calyx of Held in brainstem slices that were cut at P8-9 from mice that had been stereotactically injected at P0 with lentiviruses expressing eGFP-tagged Cre (red) or GFP only (ΔCre) (black). Sample traces (left) and summary data (right) of EPSCs. AMPAR-mediated EPSCs were recorded in the presence of NMDAR blocker APV. (**C**), Schematic of the strategy of conditional *Nlgn3* deletion from the calyx of Held synapse. (**D**), Example traces of AMPAR-mediated EPSCs recording from P12/13 MNTB neurons (control, black; *Nlgn1/3* KO, red). (**E**), Analyses of EPSC amplitude (left), rise time (middle), and decay time (right) in D. (**F**), Analysis of the coefficient of variation of EPSC in D. (**G**), Example traces of AMPAR-mediated mEPSCs recording from P12/13 MNTB neurons (control, black; *Nlgn1/3* KO, red). (**H** and **I**), Analyses of mEPSC frequency and amplitude (H), rise time, and decay time (I).

Data are means ± SEM. Numbers in bars represent the numbers of cells/animals. Statistical significance was determined by a two-tailed Student's *t*-test (B, E, F, H, and I), or by the Kolmogorov-Smirnov test (H), with $^{*}P < 0.05$, $^{**}P < 0.01$, $^{***}P < 0.001$.

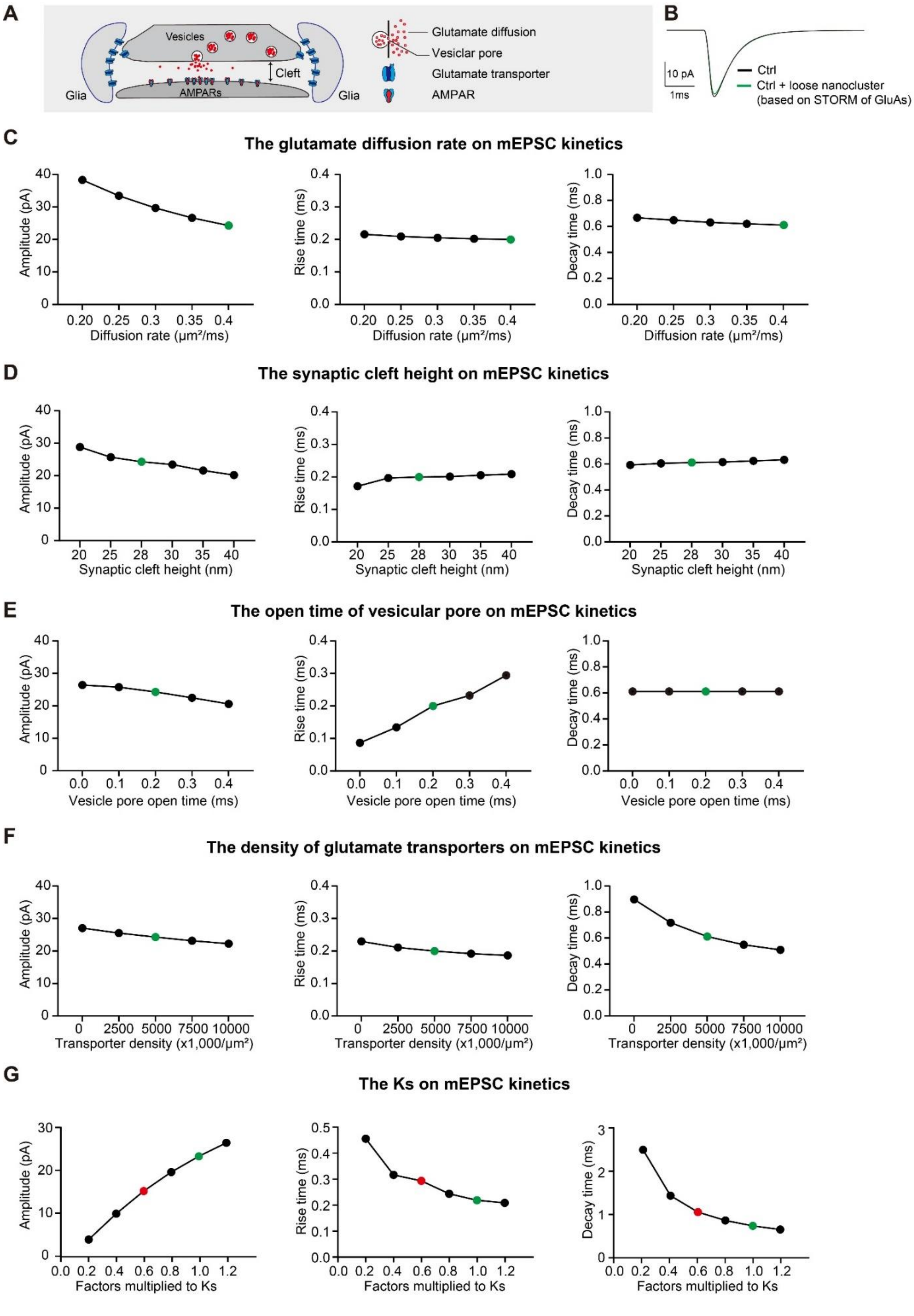

A
Vesicles
Cleft
AMPARs
Glia
Glia
Glutamate diffusion
Vesiclar pore
Glutamate transporter
AMPAR
B
10 pA
1ms
Ctrl
Ctrl + loose nanocluster
(based on STORM of GluAs)
C
The glutamate diffusion rate on mEPSC kinetics
Amplitude (pA)
Rise time (ms)
Decay time (ms)
Diffusion rate (μm²/ms)
D
The synaptic cleft height on mEPSC kinetics
Synaptic cleft height (nm)
E
The open time of vesicular pore on mEPSC kinetics
Vesicle pore open time (ms)
F
The density of glutamate transporters on mEPSC kinetics
Transporter density (x1,000/μm²)
G
The Ks on mEPSC kinetics
Factors multiplied to Ks

fig. S2.

Simulations of potential parameters that might affect mEPSC kinetics.

(**A**), Three-dimensional synaptic geometry used in the simulations of mEPSC at the calyx of Held. (**B**), Simulated mEPSC of control and Pv-*Nlgn3* at P12 based on STORM data of GluA1 and GluA4. (**C-G**), Potential parameters that may be affected by *Nlgn3* knockout at P12. Plots of amplitude, rise time, and decay time of simulated mEPSC with different diffusion rates of glutamate (C), with different cleft heights (D), with different vesicular pore open time (E), with different extrasynaptic transporter densities (F), or with different factors that multiplied to Ks (G). The sets of parameters used in simulating control and the effects of loose GluAs nanoclusters (Fig. 6D and E, black and green) were labeled as green and the factor used in simulating slower Ks (Fig. 6E, red) was labeled as red in G.

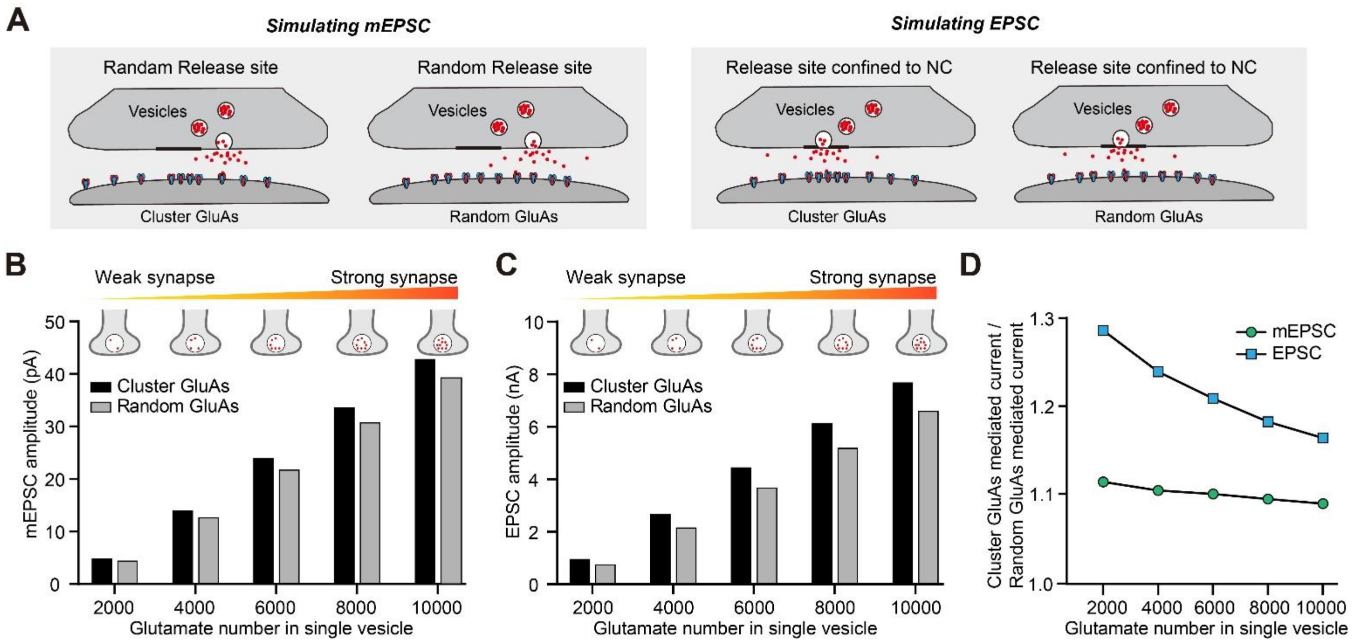

fig. S3.

Simulation of the impact of nanoclusters and the transmitter content of synaptic vesicles on mEPSCs and EPSCs.

(A), Schematic of simulations of AMPAR-mediated mEPSC and EPSC. AMPAR-mediated mEPSC was simulated by randomized release sites (left panel) against the cluster GluAs and random GluAs, respectively. For EPSC, the release sites were constrained to the nanocluster which is aligned to the postsynaptic nanocluster (right panel). NC: nanocluster. **(B)**, The amplitude of simulated mEPSC for both cluster GluAs and random GluAs. The varied glutamate number denotes the weak and strong synapses. **(C)**, The amplitude of simulated EPSC for both cluster GluAs and random GluAs. **(D)**, The ratio of current mediated by the cluster GluAs to the random GluAs. All simulations were an average of 160 runs. Also, see details in the Methods.

table S1.
Default parameters used for simulation.

Time step size (*9*)	0.5 μs
Cleft height (*79*)	28 nm
Vesicle fusion duration (*74*, *78*)	0.2 ms
AMPAR number (*44*, *79*)	100
Glutamate diffusion rate (*44*)	0.4 $\mu m^2/ms$
Conductance (*13*)	31 pS for slow-GluAs; 45 pS for fast-GluAs
Distance between synapse and glial sheath (*9*)	40 nm

table S2.

Parameters used in Fig. 6B and C and fig. S2B (based on GluA1/GluA4 STORM data)

		**P4**	**P8**	**P12-Ctrl**	**P12- KO**	**P16**	**P30**
Radiuses based on EM (*73*) and STORM (nm) [a]	GluA1 (slow)	SC: 173.6<br>NC: 51.1	SC: 173.6<br>NC: 51.1	SC: 139.7<br>NC: 41.1	SC: 129.6<br>NC: 45.6	SC: 139.7<br>NC: 41.1	SC: 139.7<br>NC: 41.1
	GluA4 (fast)	SC: 153.8<br>NC: 42.2	SC: 153.8<br>NC: 42.2	SC: 123.8<br>NC: 34.0	SC: 134.4<br>NC: 37.6	SC: 123.8<br>NC: 34.0	SC: 123.8<br>NC: 34.0
Radius of the active zone (nm) [b]		163.7	163.7	131.8	132.0	131.8	131.8
GluAs number based on STORM	GluA1	SC: 70<br>NC: 15	SC: 58<br>NC: 12	SC: 37<br>NC: 8	SC: 37<br>NC: 8	SC: 35<br>NC: 7	SC: 21<br>NC: 4
	GluA4	SC: 13<br>NC: 2	SC: 26<br>NC: 4	SC: 47<br>NC: 8	SC: 46<br>NC: 9	SC: 50<br>NC: 8	SC: 64<br>NC: 11
Glutamate per vesicle (*53*, *54*)		8000	8000	8000	8000	10000	10000
Transporter densities (*9*)		5000 /μm^2	5000 /μm^2	5000 /μm^2	5000 /μm^2	8000 /μm^2	8000 /μm^2
Ratios in different stages (fast-GluAs / All GluAs) (*15*, *52*)		15%	30%	55%	55%	58%	75%

a. The EM data is further calculated by STORM data at P12, see details in Methods: Simulations and codes. 164 nm for P4 and P8, and 132 nm for P12, P16, and P30 as indicated in EM (73). SC, synaptic cluster. NC, nanocluster.
b. The active zone has the same radius as the average of GluA1/A4 synaptic clusters.
More details can be referred to the computational model on Zenodo (https://doi.org/10.5281/zenodo.6419982) or on GitHub (https://github.com/Han-y/Model-of-Synapse.git).

table S3.

Parameters used in Fig. 6D (based on PSD-95 STORM data).

		**Ctrl**	**Ctrl + loose GluAs**	**Ctrl + loose GluAs + small Ks**
GluAs number in each PSD-95 subregion [a]	0-60 nm	slow-GluAs: 16.1, fast-GluAs: 19.6	slow-GluAs: 10, fast-GluAs: 12.2	slow-GluAs: 10, fast-GluAs: 12.2
	60-100 nm:	slow-GluAs: 13.9, fast-GluAs: 17	slow-GluAs: 14.9, fast-GluAs: 18.1	slow-GluAs: 14.9, fast-GluAs: 18.1
	100-140 nm:	slow-GluAs: 15, fast-GluAs: 18.4	slow-GluAs: 17.4, fast-GluAs: 21.2	slow-GluAs: 17.4, fast-GluAs: 21.2
Factor (*44*, *79*)		1	1	0.6
The radius of the PSD-95 synaptic cluster		140 nm		
The radius of RIM synaptic cluster [b]		140 nm		
Glutamate per vesicle (*53*, *54*)		8000		
Transporter densities (*9*)		5000 /μm^2		

a. The numbers of receptors do not need to be integers, because the simulation could be regarded as an average of a series of reactions with integral receptor numbers.

b. The active zone is the same as PSD-95 nanoclusters (60 nm) when simulating AMPAR-mediated EPSCs and as PSD-95 synaptic cluster (140 nm) when simulating AMPAR-mediated mEPSCs in fig. S3.

More details can be referred to the computational model on Zenodo (https://doi.org/10.5281/zenodo.6419982) or on GitHub (https://github.com/Han-y/Model-of-Synapse.git).

table S4.

Rate constants for slow-GluAs and fast-GluAs

Rate constants	Slow-GluAs	Fast-GluAs
K_1	1.5×10^7	2.2×10^7
K_{-1}	4.323×10^3	4.7553×10^3
K_2	4×10^6	4.4×10^6
K_{-2}	1.7201×10^4	1.89211×10^4
K_3	1.0×10^4	2.75×10^4
K_{-3}	2.7×10^3	8.8×10^3
K_4	1	1.1×10^2
K_{-4}	0.42183	115.995
K_5	1.9863×10^7	2.18493×10^7
K_{-5}	1.8392×10^4	15.17307×10^3
K_6	8.48141	310.2
K_{-6}	12.13	533.555
K_7	25.85	56.87
K_{-7}	93.287	51.3084
K_8	1.1813×10^3	974.589
K_{-8}	280.35	308.385
K_9	380.434	418.4774
K_{-9}	19.44	21.384
K_{10}	0.59666	6.56326
K_{-10}	0.17436	1.91796
K_{11}	2	2200
K_{-11}	5	550

K_1, K_2 and K_5 have units of $M^{-1}s^{-1}$ and s^{-1} for the rest.

REFERENCES AND NOTES

1. G. H. Diering, R. L. Huganir, The AMPA receptor code of synaptic plasticity. *Neuron* **100**, 314–329 (2018).

2. L. Groc, D. Choquet, Linking glutamate receptor movements and synapse function. *Science* **368**, eaay4631 (2020).

3. T. Biederer, P. S. Kaeser, T. A. Blanpied, Transcellular nanoalignment of synaptic function. *Neuron* **96**,680–696 (2017).

4. H. D. MacGillavry, Y. Song, S. Raghavachari, T. A. Blanpied, Nanoscale scaffolding domains within the postsynaptic density concentrate synaptic ampa receptors. *Neuron* **78**, 615–622 (2013).

5. M. Masugi-Tokita, E. Tarusawa, M. Watanabe, E. Molnar, K. Fujimoto, R. Shigemoto, Number and density of AMPA receptors in individual synapses in the rat cerebellum as revealed by SDS-digested freeze-fracture replica labeling. *J. Neurosci.* **27**, 2135–2144 (2007).

6. D. Nair, E. Hosy, J. D. Petersen, A. Constals, G. Giannone, D. Choquet, J. B. Sibarita, Super-resolution imaging reveals that AMPA receptors inside synapses are dynamically organized in nanodomains regulated by PSD95. *J. Neurosci.* **33**, 13204–13224 (2013).

7. A. M. Ramsey, A.-H. Tang, T. A. Le Gates, X.-Z. Gou, B. E. Carbone, S. M. Thompson, T. Biederer, T. A. Blanpied, Subsynaptic positioning of AMPARs by LRRTM2 controls synaptic strength. *Sci. Adv.* **7**, eabf3126 (2021).

8. A. H. Tang, H. Chen, T. P. Li, S. R. Metzbower, H. D. MacGillavry, T. A. Blanpied, A trans-synaptic nanocolumn aligns neurotransmitter release to receptors. *Nature* **536**, 210–214 (2016).

9. D. Freche, U. Pannasch, N. Rouach, D. Holcman, Synapse geometry and receptor dynamics modulate synaptic strength. *PLOS ONE* **6**, eabf3126 (2011).

10. D. A. Rusakov, The role of perisynaptic glial sheaths in glutamate spillover and extracellular $Ca2^{+}$ depletion. *Biophys. J.* **81**, 1947–1959 (2001).

11. D. A. Rusakov, D. M. Kullmann, Extrasynaptic glutamate diffusion in the hippocampus: Ultrastructural constraints, uptake, and receptor activation. *J. Neurosci.* **18**, 3158–3170 (1998).

12. E. Tarusawa, K. Matsui, T. Budisantoso, E. Molnar, M. Watanabe, M. Matsui, Y. Fukazawa, R. Shigemoto, Input-specific intrasynaptic arrangements of ionotropic glutamate receptors and their impact on postsynaptic responses. *J. Neurosci.* **29**, 12896–12908 (2009).

13. S. F. Traynelis, L. P. Wollmuth, C. J. McBain, F. S. Menniti, K. M. Vance, K. K. Ogden, K. B. Hansen, H. Yuan, S. J. Myers, R. Dingledine, Glutamate receptor ion channels: Structure, regulation, and function. *Pharmacol. Rev.* **62**, 405–496 (2010).

14. W. Lu, Y. Shi, A. C. Jackson, K. Bjorgan, M. J. During, R. Sprengel, P. H. Seeburg, R. A. Nicoll, Subunit composition of synaptic AMPA receptors revealed by a single-cell genetic approach. *Neuron* **62**, 254–268 (2009).

15. Y. M. Yang, J. Aitoubah, A. M. Lauer, M. Nuriya, K. Takamiya, Z. Jia, B. J. May, R. L. Huganir, L. Y. Wang, GluA4 is indispensable for driving fast neurotransmission across a high-fidelity central synapse. *J. Physiol.* **589**, 4209–4227 (2011).

16. T. C. Südhof, Synaptic neurexin complexes: A molecular code for the logic of neural circuits. *Cell* **171**, 745–769 (2017).

17. S. Jamain, H. Quach, C. Betancur, M. Råstam, C. Colineaux, I Carina Gillberg, H. Soderstrom, B. Giros, M. Leboyer, C. Gillberg, T. Bourgeron; Paris Autism Research International Sibpair Study, Mutations of the X-linked genes encoding neuroligins NLGN3 and NLGN4 are associated with autism. *Nat. Genet.* **34**, 27–29 (2003).

18. M. Nakanishi, J. Nomura, X. Ji, K. Tamada, T. Arai, E. Takahashi, M. Bućan, T. Takumi, Functional significance of rare neuroligin 1 variants found in autism. *PLOS Genet.* **13**, e1006940 (2017).

19. T. A. Nguyen, K. Wu, S. Pandey, A. W. Lehr, Y. Li, M. A. Bemben, John D Badger II, J. L. Lauzon, T. Wang, K. A. Zaghloul, A. Thurm, M. Jain, W. Lu, K. W. Roche, A cluster of autism-

associated variants on x-linked NLGN4X functionally resemble NLGN4Y. *Neuron* **106**, 759–768.e7 (2020).

20. A. Quartier, J. Courraud, T. Thi Ha, G. McGillivray, B. Isidor, K. Rose, N. Drouot, M. A. Savidan, C. Feger, H. Jagline, J. Chelly, M. Shaw, F. Laumonnier, J. Gecz, J. L. Mandel, A. Piton, Novel mutations in NLGN3 causing autism spectrum disorder and cognitive impairment. *Hum. Mutat.* **40**, 2021–2032 (2019).

21. A. M. Craig, Y. Kang, Neurexin-neuroligin signaling in synapse development. *Curr. Opin. Neurobiol.* **17**, 43–52 (2007).

22. M. Hoon, T. Soykan, B. Falkenburger, M. Hammer, A. Patrizi, K. F. Schmidt, M. Sassoè-Pognetto, S. Löwel, T. Moser, H. Taschenberger, N. Brose, F. Varoqueaux, Neuroligin-4 is localized to glycinergic postsynapses and regulates inhibition in the retina. *Proc. Natl. Acad. Sci. U.S.A.* **108**, 3053–3058 (2011).

23. D. Krueger-Burg, T. Papadopoulos, N. Brose, Organizers of inhibitory synapses come of age. *Curr. Opin. Neurobiol.* **45**, 66–77 (2017).

24. K. Nozawa, A. Hayashi, J. Motohashi, Y. H. Takeo, K. Matsuda, M. Yuzaki, Cellular and subcellular localization of endogenous neuroligin-1 in the cerebellum. *Cerebellum* **17**, 709–721 (2018).

25. A. Poulopoulos, G. Aramuni, G. Meyer, T. Soykan, M. Hoon, T. Papadopoulos, M. Zhang, I. Paarmann, C. Fuchs, K. Harvey, P. Jedlicka, S. W. Schwarzacher, H. Betz, R. J. Harvey, N. Brose, W. Zhang, F. Varoqueaux, Neuroligin 2 drives postsynaptic assembly at perisomatic inhibitory synapses through gephyrin and collybistin. *Neuron* **63**, 628–642 (2009).

26. T. C. Südhof, Towards an understanding of synapse formation. *Neuron* **100**, 276–293 (2018).

27. W. Cao, S. Lin, Q.-Q. Xia, Y.-L. Du, Q. Yang, M.-Y. Zhang, Y.-Q. Lu, J. Xu, S.-M. Duan, J. Xia, G. Feng, J. Xu, J.-H. Luo, Gamma oscillation dysfunction in mPFC leads to social deficits in neuroligin 3 R451C knockin mice. *Neuron* **97**, 1253–1260.e7 (2018).

28. M. Jiang, J. Polepalli, L. Y. Chen, B. Zhang, T. C. Südhof, R. C. Malenka, Conditional ablation of neuroligin-1 in CA1 pyramidal neurons blocks LTP by a cell-autonomous NMDA receptor-independent mechanism. *Mol. Psychiatry* **22**, 375–383 (2017).

29. S. Y. Jung, J. Kim, O. B. Kwon, J. H. Jung, K. An, A. Y. Jeong, C. J. Lee, Y. B. Choi, C. H. Bailey, E. R. Kandel, J. H. Kim, Input-specific synaptic plasticity in the amygdala is regulated by neuroligin-1 via postsynaptic NMDA receptors. *Proc. Natl. Acad. Sci. U.S.A.* **107**, 4710–4715 (2010).

30. J. S. Polepalli, H. Wu, D. Goswami, C. H. Halpern, T. C. Südhof, R. C. Malenka, Modulation of excitation on parvalbumin interneurons by neuroligin-3 regulates the hippocampal network. *Nat. Neurosci.* **20**, 219–229 (2017).

31. E. Troyano-Rodriguez, C. R. Wirsig-Wiechmann, M. Ahmad, Neuroligin-2 determines inhibitory synaptic transmission in the lateral septum to optimize stress-induced neuronal activation and avoidance behavior. *Biol. Psychiatry* **85**, 1046–1055 (2019).

32. X. Wu, W. K. Morishita, A. M. Riley, W. D. Hale, T. C. Südhof, R. C. Malenka, Neuroligin-1 signaling controls LTP and NMDA receptors by distinct molecular pathways. *Neuron* **102**, 621–635.e3 (2019).

33. B. Zhang, E. Seigneur, P. Wei, O. Gokce, J. Morgan, T. C. Südhof, Developmental plasticity shapes synaptic phenotypes of autism-associated neuroligin-3 mutations in the calyx of Held. *Mol. Psychiatry* **22**, 1483–1491 (2017).

34. M. Uchigashima, K. Konno, E. Demchak, A. Cheung, T. Watanabe, D. G. Keener, M. Abe, T. le, K. Sakimura, T. Sasaoka, T. Uemura, Y. Imamura Kawasawa, M. Watanabe, K. Futai, Specific Neuroligin3-αNeurexin1 signaling regulates GABAergic synaptic function in mouse hippocampus. *eLife* **9,** e59545 (2020).

35. M. Uchigashima, M. Leung, T. Watanabe, A. Cheung, T. le, S. Pallat, A. L. M. Dinis, M. Watanabe, Y. I. Kawasawa, K. Futai, Neuroligin3 splice isoforms shape inhibitory synaptic function in the mouse hippocampus. *J. Biol. Chem.* **295**, 8589–8595 (2020).

36. K. T. Haas, B. Compans, M. Letellier, T. M. Bartol, D. Grillo-Bosch, T. J. Sejnowski, M. Sainlos, D. Choquet, O. Thoumine, E. Hosy, Pre-post synaptic alignment through neuroligin-1 tunes synaptic transmission efficiency. *eLife* **7**, e31755 (2018).

37. A. A. Chubykin, D. Atasoy, M. R. Etherton, N. Brose, E. T. Kavalali, J. R. Gibson, T. C. Südhof, Activity-dependent validation of excitatory versus inhibitory synapses by neuroligin-1 versus neuroligin-2. *Neuron* **54**, 919–931 (2007).

38. S. Chanda, W. D. Hale, B. Zhang, M. Wernig, T. C. Südhof, Unique versus redundant functions of neuroligin genes in shaping excitatory and inhibitory synapse properties. *J. Neurosci.* **37**, 6816–6836 (2017).

39. C. Acuna, Q. Guo, J. Burré, M. Sharma, J. Sun, T. C. Südhof, Microsecond dissection of neurotransmitter release: SNARE-complex assembly dictates speed and $Ca2^{+}$ sensitivity. *Neuron* **82**, 1088–1100 (2014).

40. I. Joshi, L. Y. Wang, Developmental profiles of glutamate receptors and synaptic transmission at a single synapse in the mouse auditory brainstem. *J. Physiol.* **540**, 861–873 (2002).

41. T. Budisantoso, H. Harada, N. Kamasawa, Y. Fukazawa, R. Shigemoto, K. Matsui, Evaluation of glutamate concentration transient in the synaptic cleft of the rat calyx of Held. *J. Physiol.* **591**, 219–239 (2013).

42. N. Chuhma, H. Ohmori, Postnatal development of phase-locked high-fidelity synaptic transmission in the medial nucleus of the trapezoid body of the rat. *J. Neurosci.* **18**, 512–520 (1998).

43. J. Y. Song, K. Ichtchenko, T. C. Südhof, N. Brose, Neuroligin 1 is a postsynaptic cell-adhesion molecule of excitatory synapses. *Proc. Natl. Acad. Sci. U.S.A.* **96**, 1100–1105 (1999).

44. S. Raghavachari, J. E. Lisman, Properties of quantal transmission at CA1 synapses. *J. Neurophysiol.* **92**, 2456–2467 (2004).

45. A. Dani, B. Huang, J. Bergan, C. Dulac, X. Zhuang, Superresolution imaging of chemical synapses in the brain. *Neuron* **68**, 843–856 (2010).

46. P. S. Kaeser, L. Deng, Y. Wang, I. Dulubova, X. Liu, J. Rizo, T. C. Südhof, RIM proteins tether $Ca2^{+}$ channels to presynaptic active zones via a direct PDZ-domain interaction. *Cell* **144**, 282–295 (2011).

47. J. H. Chen, T. A. Blanpied, A. H. Tang, Quantification of trans-synaptic protein alignment: A data analysis case for single-molecule localization microscopy. *Methods* **174**, 72–80 (2020).

48. C. Acuna, X. Liu, T. C. Südhof, How to make an active zone: Unexpected universal functional redundancy between RIMs and RIM-BPs. *Neuron* **91**, 792–807 (2016).

49. M. M. Brockmann, F. Zarebidaki, M. Camacho, M. K. Grauel, T. Trimbuch, T. C. Südhof, C. Rosenmund, A trio of active zone proteins comprised of RIM-BPs, RIMs, and Munc13s governs neurotransmitter release. *Cell Rep.* **32**, 107960 (2020).

50. O. R. Buonarati, E. A. Hammes, J. F. Watson, I. H. Greger, J. W. Hell, Mechanisms of postsynaptic localization of AMPA-type glutamate receptors and their regulation during long-term potentiation. *Sci. Signal.* **12**, eaar6889 (2019).

51. P. Opazo, M. Sainlos, D. Choquet, Regulation of AMPA receptor surface diffusion by PSD-95 slots. *Curr. Opin. Neurobiol.* **22**, 453–460 (2012).

52. B. Lujan, A. Dagostin, H. Von Gersdorff, Presynaptic diversity revealed by $Ca2^{+}$-permeable AMPA receptors at the calyx of held synapse. *J. Neurosci.* **39**, 2981–2994 (2019).

53. J. Guo, Z. C. Sun, P. T. Yao, H. L. Wang, L. Xue, A Monte Carlo simulation dissecting quantal release at the calyx of Held. *Front. Biosci.* **20**, 1079–1091 (2015).

54. T. Yamashita, T. Ishikawa, T. Takahashi, Developmental increase in vesicular glutamate content does not cause saturation of AMPA receptors at the calyx of Held synapse. *J. Neurosci.* **23**, 3633–3638 (2003).

55. M. Heine, D. Holcman, Asymmetry between pre- and postsynaptic transient nanodomains shapes neuronal communication. *Trends Neurosci.* **43**, 182–196 (2020).

56. M. J. Fedchyshyn, L. Y. Wang, Developmental transformation of the release modality at the calyx of held synapse. *J. Neurosci.* **25**, 4131–4140 (2005).

57. Y. Nakamura, H. Harada, N. Kamasawa, K. Matsui, J. S. Rothman, R. Shigemoto, R. A. Silver, D. A. DiGregorio, T. Takahashi, Nanoscale distribution of presynaptic $Ca2^+$ channels and its impact on vesicular release during development. *Neuron* **85**, 145–158 (2015).

58. Y.-M. Yang, M. J. Fedchyshyn, G. Grande, J. Aitoubah, C. W. Tsang, H. Xie, C. A. Ackerley, W. S. Trimble, L. Y. Wang, Septins regulate developmental switching from microdomain to nanodomain coupling of Ca(2+) influx to neurotransmitter release at a central synapse. *Neuron* **67**, 100–115 (2010).

59. K. Futai, M. Okada, K. Matsuyama, T. Takahashi, High-fidelity transmission acquired via a developmental decrease in NMDA receptor expression at an auditory synapse. *J. Neurosci.* **21**, 3342–3349 (2001).

60. H. Taschenberger, H. Von Gersdorff, Fine-tuning an auditory synapse for speed and fidelity: Developmental changes in presynaptic waveform, EPSC kinetics, and synaptic plasticity. *J. Neurosci.* **20**, 9162–9173 (2000).

61. P. E. Rothwell, M. V. Fuccillo, S. Maxeiner, S. J. Hayton, O. Gokce, B. K. Lim, S. C. Fowler, R. C. Malenka, T. C. Südhof, Autism-associated neuroligin-3 mutations commonly impair striatal circuits to boost repetitive behaviors. *Cell* **158**, 198–212 (2014).

62. M. Irie, Y. Hata, M. Takeuchi, K. Ichtchenko, A. Toyoda, K. Hirao, Y. Takai, T. W. Rosahl, T. C. Südhof, Binding of neuroligins to PSD-95. *Science* **277**, 1511–1515 (1997).

63. J. García-Nafría, B. Herguedas, J. F. Watson, I. H. Greger, The dynamic AMPA receptor extracellular region: A platform for synaptic protein interactions. *J. Physiol.* **594**, 5449–5458 (2016).

64. C. I. Nam, L. Chen, Postsynaptic assembly induced by neurexin-neuroligin interaction and neurotransmitter. *Proc. Natl. Acad. Sci. U.S.A.* **102**, 6137–6142 (2005).

65. M. Zeng, X. Chen, D. Guan, J. Xu, H. Wu, P. Tong, M. Zhang, Reconstituted postsynaptic density as a molecular platform for understanding synapse formation and plasticity. *Cell* **174**, 1172–1187.e16 (2018).

66. E. C. Budreck, P. Scheiffele, Neuroligin-3 is a neuronal adhesion protein at GABAergic and glutamatergic synapses. *Eur. J. Neurosci.* **26**, 1738–1748 (2007).

67. C. Földy, R. C. Malenka, T. C. Südhof, Autism-associated neuroligin-3 mutations commonly disrupt tonic endocannabinoid signaling. *Neuron* **78**, 498–509 (2013).

68. B. Zhang, L. Y. Chen, X. Liu, S. Maxeiner, S. J. Lee, O. Gokce, T. C. Südhof, Neuroligins sculpt cerebellar purkinje-cell circuits by differential control of distinct classes of synapses. *Neuron* **87**, 781–796 (2015).

69. J. Dai, C. Patzke, K. Liakath-Ali, E. Seigneur, T. C. Südhof, GluD1 is a signal transduction device disguised as an ionotropic receptor. *Nature* **595**, 261–265 (2021).

70. I. H. Greger, J. F. Watson, S. G. Cull-Candy, Structural and functional architecture of AMPA-type glutamate receptors and their auxiliary proteins. *Neuron* **94**, 713–730 (2017).

71. D. M. O. Ramirez, E. T. Kavalali, Differential regulation of spontaneous and evoked neurotransmitter release at central synapses. *Curr. Opin. Neurobiol.* **21**, 275–282 (2011).

72. S. Hippenmeyer, E. Vrieseling, M. Sigrist, T. Portmann, C. Laengle, D. R. Ladle, S. Arber, A developmental switch in the response of DRG neurons to ETS transcription factor signaling. *PLOS Biol.* **3**, 0878–0890 (2005).

73. H. Taschenberger, R. M. Leão, K. C. Rowland, G. A. Spirou, H. Von Gersdorff, Optimizing synaptic architecture and efficiency for high-frequency transmission. *Neuron* **36**, 1127–1143 (2002).

74. L. He, X. S. Wu, R. Mohan, L. G. Wu, Two modes of fusion pore opening revealed by cell-attached recordings at a synapse. *Nature* **444**, 102–105 (2006).

75. L. W. Gong, G. A. de Toledo, M. Lindau, Exocytotic catecholamine release is not associated with cation flux through channels in the vesicle membrane but Na^+ influx through the fusion pore. *Nat. Cell Biol.* **9**, 915–922 (2007).

76. D. Colquhoun, K. A. Dowsland, M. Beato, A. J. R. Plested, How to impose microscopic reversibility in complex reaction mechanisms. *Biophys. J.* **86**, 3510–3518 (2004).

77. D. Hermida, J. M. Mateos, I. Elezgarai, N. Puente, A. Bilbao, J. L. Bueno-López, P. Streit, P. Grandes, Spatial compartmentalization of AMPA glutamate receptor subunits at the calyx of held synapse. *J. Comp. Neurol.* **518**, 163–174 (2010).

78. C. W. Chang, C. W. Chiang, M. B. Jackson, Fusion pores and their control of neurotransmitter and hormone release. *J. Gen. Physiol.* **149**, 301–322 (2017).

79. M. Postlethwaite, M. H. Hennig, J. R. Steinert, B. P. Graham, I. D. Forsythe, Acceleration of AMPA receptor kinetics underlies temperature-dependent changes in synaptic strength at the rat calyx of Held. *J. Physiol.* **579**, 69–84 (2007).

80. P. L. Greer, R. Hanayama, B. L. Bloodgood, A. R. Mardinly, D. M. Lipton, S. W. Flavell, T. K. Kim, E. C. Griffith, Z. Waldon, R. Maehr, H. L. Ploegh, S. Chowdhury, P. F. Worley, J. Steen, M. E. Greenberg, The angelman syndrome protein Ube3A regulates synapse development by ubiquitinating arc. *Cell* **140**, 704–716 (2010).
